# FGFR signaling regulates resistance of head and neck cancer stem cells to cisplatin

**DOI:** 10.18632/oncotarget.25358

**Published:** 2018-05-18

**Authors:** Sarah C. McDermott, Christie Rodriguez-Ramirez, Sean P. McDermott, Max S. Wicha, Jacques E. Nör

**Affiliations:** ^1^ Department of Orthodontics and Pediatric Dentistry, University of Michigan School of Dentistry, Ann Arbor, MI, USA; ^2^ Department of Cariology, Restorative Science & Endodontics, University of Michigan School of Dentistry, Ann Arbor, MI, USA; ^3^ Department of Internal Medicine–Hematology/Oncology, University of Michigan Medical School, Ann Arbor, MI, USA; ^4^ Department of Biomedical Engineering, University of Michigan College of Engineering, Ann Arbor, MI, USA; ^5^ Department of Otolaryngology, University of Michigan Medical School, Ann Arbor, MI, USA

**Keywords:** chemotherapy, microarray, chemoresistance, head and neck squamous cell carcinoma, tumor-initiating cells

## Abstract

Patients with recurrent or metastatic head and neck squamous cell carcinoma (HNSCC) have poor prognosis with less than 1-year median survival. Platinum-based chemotherapy remains the first-line treatment for HNSCC. The cancer stem cell (CSC) hypothesis postulates that tumors are maintained by a self-renewing CSC population that is also capable of differentiating into non-self renewing cell populations that constitute the bulk of the tumor. A small population of CSC exists within HNSCC that are relatively resistant to chemotherapy and clinically predicted to contribute to tumor recurrence. These head and neck CSCs (HNCSC) are identified by high cell-surface expression of CD44 and high intracellular activity of aldehyde dehydrogenase (ALDH) and termed ALDH^high^CD44^high^. Here, we performed microarray analysis in two HNSCC cell lines (UM-SCC-1, UM-SCC-22B) to investigate molecular pathways active in untreated and cisplatin-resistant ALDH^high^CD44^high^ cells. Gene set enrichment analysis and iPathway analysis identified signaling pathways with major implications to the pathobiology of cancer (e.g. TNFα, IFN, IL6/STAT, NF-κB) that are enriched in cisplatin-resistant ALDH^high^CD44^high^ cells, when compared to control cells. FGF2 was also enriched in cisplatin-resistant ALDH^high^CD44^high^, which was confirmed by ELISA analysis. Inhibition of FGF signaling using BGJ398, a pan-FGF receptor (FGFR) small-molecule inhibitor, decreased ALDH^high^CD44^high^ alone in UM-SCC-1 and preferentially targeted cisplatin-resistant ALDH^high^CD44^high^ cells in UM-SCC-22B. These findings suggest that FGFR signaling might play an important role in the resistance of head and neck CSC to cisplatin. Collectively, this work suggests that some head and neck cancer patients might benefit from the combination of cisplatin and a FGFR inhibitor.

## INTRODUCTION

Conventional wisdom in cancer treatment considers mass reduction the standard for evaluating success. Less thought is given to underlying tumor cell heterogeneity, yet several recent studies have found significant heterogeneity among tumor cells [1–4]. The discrepancy between treatments designed to treat tumor cells as a single population and the finding of tumor cell heterogeneity may explain the considerably small decrease in overall cancer death rate (<5%), despite over $200 billion invested in cancer research since 1970. In the US, 59,340 new cases and 12,290 deaths from head and neck squamous cell carcinoma (HNSCC) are estimated in 2015 [5]. At diagnosis, 31% of cases are localized, 47% are regional, and 18% are distant. The 5-year relative survival rate, based on data from 2004–2010, for cancers in the oral cavity and pharynx is 66% for all races, but only 45% for African-Americans. The 5-year survival rate for patients presenting at diagnosis with distant disease is only 37% compared to 85% for localized and 61% for regional disease [5]. Furthermore, the median survival for patients with recurrent or metastatic HNSCC remains less than one year [6].

Platinum-based chemotherapy, cisplatin or carboplatin, is the typical first-line treatment for recurrent or metastatic HNSCC and cisplatin is typically combined with fluorouracil [6]. HNSCC can express the epidermal growth factor receptor (EGFR) and is associated with poor outcome [7]. Cetuximab, an IgG1 monoclonal antibody, inhibits ligand binding to EGFR [8] and stimulates antibody-dependent cell-mediated cytotoxicity [9]. The EXTREME study demonstrated the benefit of adding cetuximab to chemotherapy and was the first phase III trial in recurrent or metastatic HNSCC to improve overall survival since cisplatin was introduced [10]. Overall survival increased from 7.4 months with only chemotherapy to 10.1 months with chemotherapy plus cetuximab. Cetuximab is the only FDA-approved molecularly targeted therapeutic approved for HNSCC. Clearly, additional therapies are needed to improve the prognosis of patients with HNSCC.

In a traditional stochastic model of cancer, any cancer cell may reform or regrow the entirety of a tumor. The cancer stem cell (CSC) hypothesis postulates that tumors are maintained by a small self-renewing CSC population that is also capable of differentiating into non-self renewing cell populations that constitute the bulk of the tumor [11]. The ability of CSCs to generate progenitor cells, cells with decreased self-renewal capacity, ultimately generates heterogeneity within the tumor. Clinically, CSCs are predicted to mediate tumor recurrence after chemotherapy and radiation-therapy due to the relative inability of these modalities to effectively target CSCs [12]. By eliminating the CSCs through targeted therapy, we may cause natural cancer regression through loss of growth potential. Without targeted therapy, cancer relapse will occur through CSC division and proliferation [12]. Therefore, new therapies that target CSCs must be developed and combined with standard therapy to achieve a true cure.

In 2007, Prince and colleagues first reported the existence of a subpopulation of HNSCC cells, isolated by expression of the cell-surface protein CD44, in primary tumor samples [4]. Similar to other CSCs, these cells exhibited stem cell like properties of self-renewal, tumorigenesis, and differentiation into non-CSCs. Clinically, high frequency of CD44+ cells was associated with tumor grade and poor outcome [13]. Subsequent studies showed HNCSCs could be identified by expression of CD133 [14] and intracellular aldehyde dehydrogenase (ALDH) activity [15, 16]. HNCSCs can be identified by dual expression of ALDH^high^ and CD44^high^, but not all ALDH^high^CD44^high^ cells exhibit CSC activity [17, 18]. Regional and distant metastases in HNSCC correspond to poor prognosis and treatment options. CSCs are often hypothesized to be the origin of metastases, and as such, *in vitro* and *in vivo* work has shown HNSCC CD44^high^ cells have more migration, invasion and metastatic ability as compared to CD44^low^ cells [19]. HNCSCs were shown to be enriched after cisplatin or 5-FU treatment [20, 21], which is consistent with the presumed role of CSCs in mediating resistance to chemotherapy. Despite the important advancements in identifying HNCSCs, very little information exists about the molecular pathways active in HNCSCs [16], let alone the mechanisms that govern chemotherapy resistance of HNCSCs.

To facilitate the development of targeted therapies to eradicate HNCSCs, there exists a need for greater insight into the mechanisms that govern chemotherapy resistance of HNCSC. Here, we isolated cisplatin-resistant HNCSCs from a HNSCC cell line, identified pathways active in cisplatin-resistant HNCSCs by using microarray analysis, and then investigated the role of a candidate gene, FGF2, in resistance of HNCSCs to chemotherapy. These results provide a rich microarray resource of naïve and cisplatin HNCSCs and suggest that targeting FGF signaling in combination with cisplatin may eradicate HNCSCs.

## RESULTS

**Figure 1:**
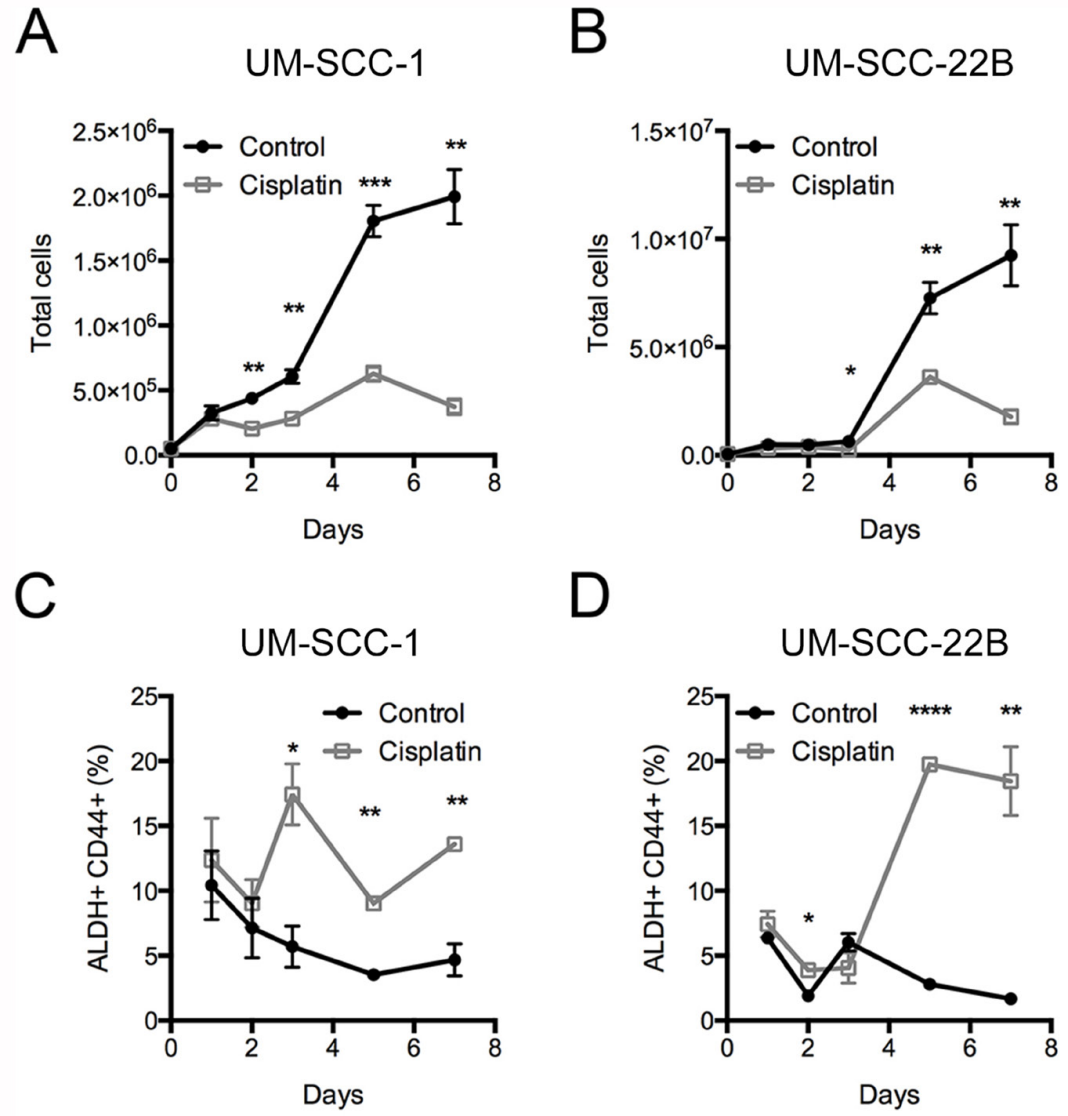
Frequency of ALDH^high^CD44^high^ cells after cisplatin treatment UM-SCC-1 and UM-SCC-22B cells were treated with control (black circles) or 2 μM cisplatin (grey open squares) for up to 7 days. The total number of cells for (**A**) UM-SCC-1 and (**B**) UM-SCC-22B. The frequency of (**C**, **D**) ALDH^high^CD44^high^ cells based on gates from DEAB sample.

To understand the chemotherapy resistance mechanisms of ALDH^high^CD44^high^ cells in HNSCC, we used two HNSCC cell lines, UM-SCC-1 and UM-SCC-22B [22]. UM-SCC-1 was from a primary tumor at the floor of the mouth, and UM-SCC-22B was from a neck metastasis derived from a tumor in the hypopharynx. The cisplatin IC_50_ for UM-SCC-1 was 1.77 ± 0.78 μM and UM-SCC-22B was higher at 5.51 ± 1.37 μM (Supplementary Figure 1). Initial experiments to examine the resistance of ALDH^high^CD44^high^ cells to cisplatin at the IC_50_ concentrations were highly variable (data not shown). Based on published reports [21], we utilized 2 μM cisplatin for additional experiments. Additional experiments at 2 μM showed maximal enrichment of ALDH^high^CD44^high^ cells in both UM-SCC-1 and UM-SCC-22B cell lines after 5 days of treatment (Figure 1, Supplementary Figures 2, 3).

**Figure 2:**
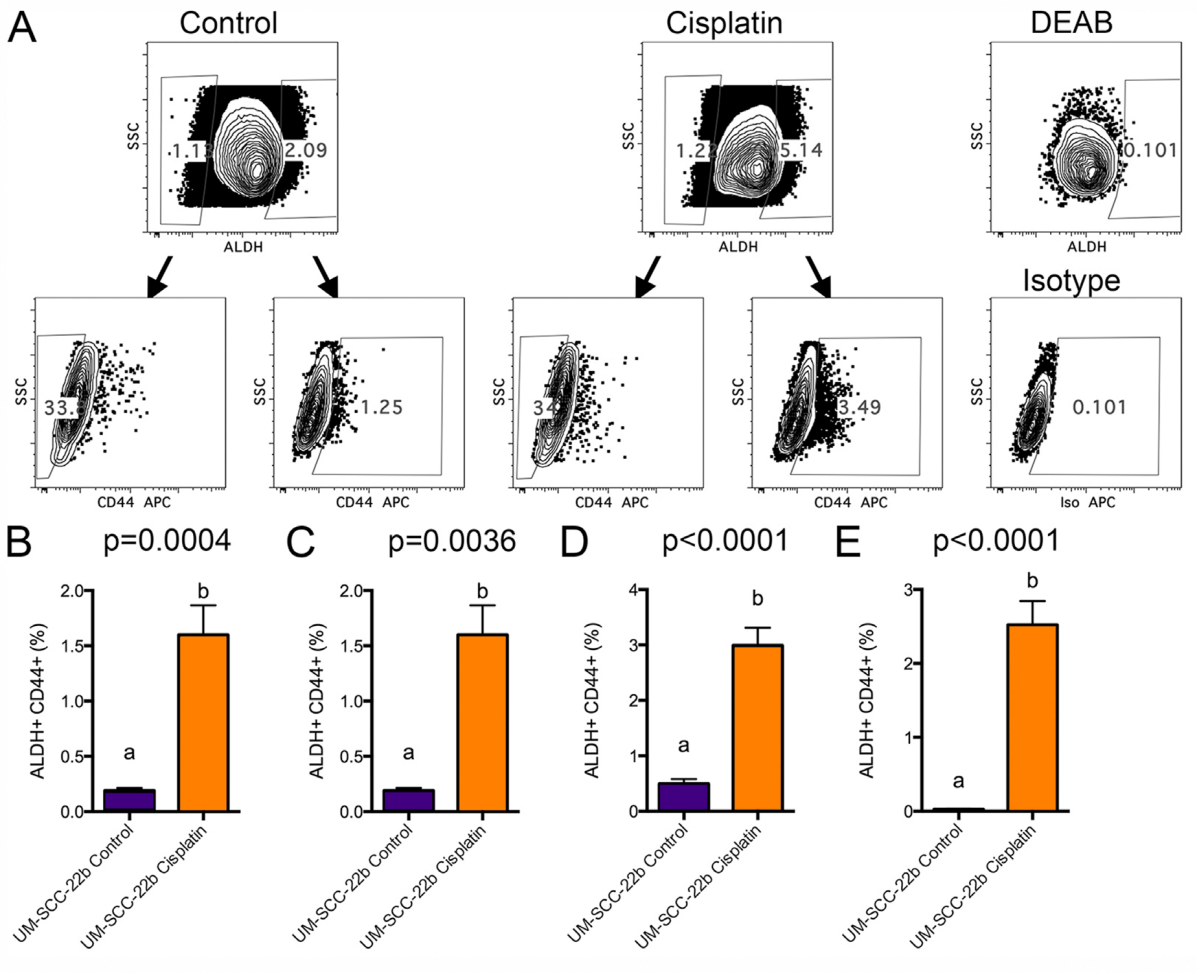
FACS analysis of cisplatin treated UM-SCC-22B cells UM-SCC-22B cells were treated for 5 days in 6-well plates with or without 2 μM cisplatin. Cells were harvested, counted, stained for ALDH and CD44, and collected by FACS. (**A**) FACS gating schema depicting how ALDH^high^CD44^high^ and ALDH^low^CD44^low^ cell populations were collected from control (left) and 2 μM cisplatin treated (right) cells. ALDH^high^ and CD44^high^ gates were set based on DEAB control FACS samples using 0.1% as a background (right). (**B**–**E**) Average ± standard deviation of ALDH^high^CD44^high^ percentage (UM-SCC-22B) for control and cisplatin groups (*N* = 6) from 4 separate FACS sorting experiments. Different letters depict statistically significant differences based on pairwise comparisons of control to cisplatin (*P <* 0.05).

To determine if 2 μM cisplatin and 5 days of treatment would provide a reasonable amount of gene expression changes, we initiated a pilot microarray experiment with UM-SCC-22B to test if it was possible to obtain a sufficient number of cells from flow cytometry sorting. ALDH^high^CD44^high^ and ALDH^low^CD44^low^ cells from control and cisplatin treated UM-SCC-22B cells were collected. The gating schema used for collecting cells by flow cytometry is shown in Figure 2A. Based on probe sets with a fold change of 2 or more with the added constraint that one of the two samples had an expression value of 2^4^ or greater, there were 234 probe sets differing between cisplatin ALDH^high^CD44^high^ and control ALDH^high^CD44^high^ cells. FGF2, EREG (epiregulin), AREG (amphiregulin), and SPRR1B (small proline-rich protein 1B) were some of the genes higher in cisplatin ALDH^high^CD44^high^.

In order to provide robust statistical comparison between cisplatin ALDH^high^CD44^high^ and control ALDH^high^CD44^high^ cells, four additional sorts of ALDH^high^CD44^high^ and ALDH^low^CD44^low^ populations were performed (Figure 2B–2E). In general, very few ALDH^high^CD44^high^ cells were collected (Supplementary Table 1). Even though there was limiting total RNA in the ALDH^high^CD44^high^ fractions (average 113 ng), RNA quality, as determined by Bioanalyzer analysis, ranged from 7.8 to 10.0, which was robust enough for microarray analysis. Due to limited RNA for experiment #4, RNA from experiments 1–3 were utilized for microarray analysis using at least 10 ng of total RNA processed with the Ovation Pico whole transcriptome amplification kit and hybridized with Affymetrix Human Gene ST 2.1 plates.

**Figure 3:**
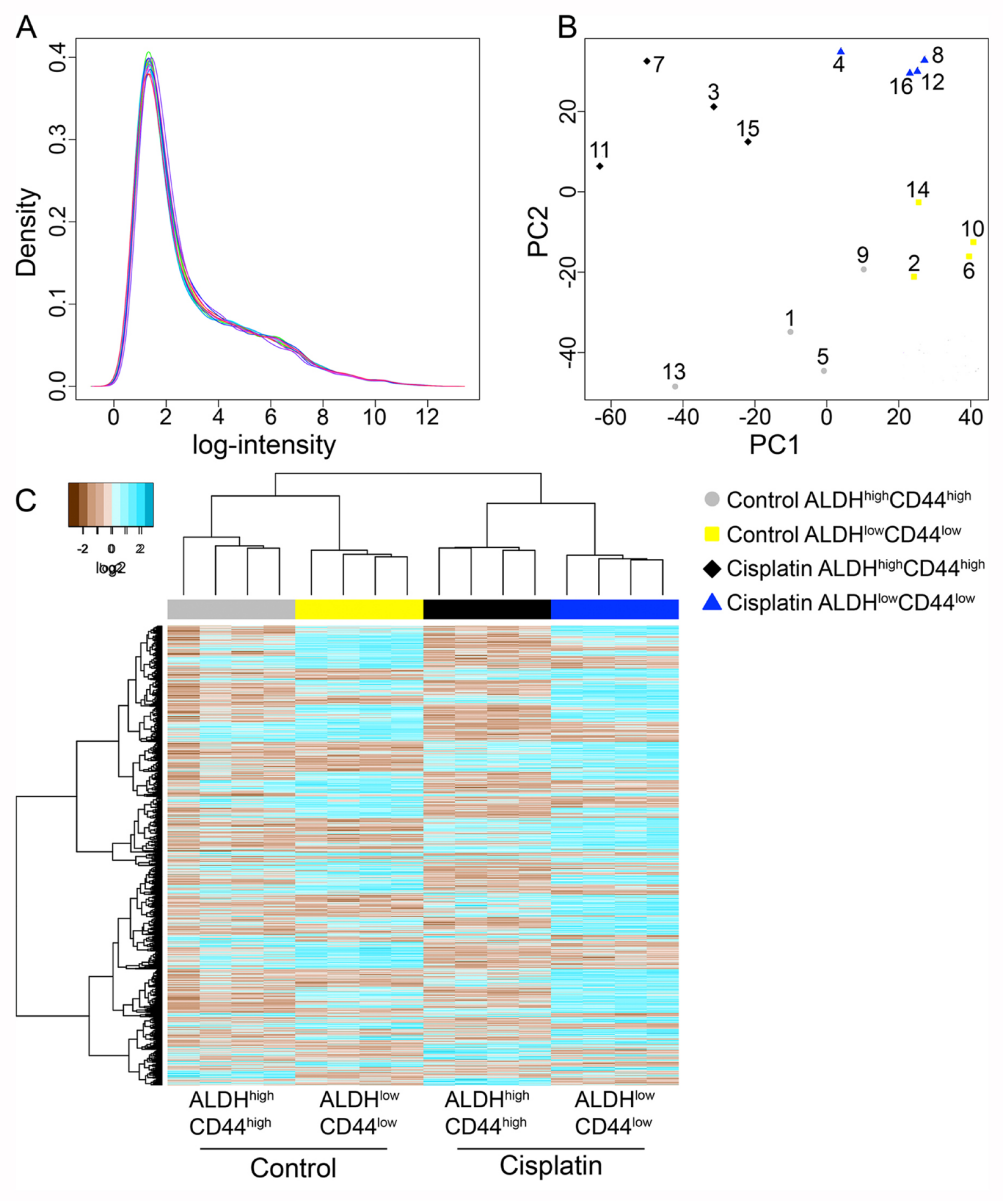
Microarray analysis of ALDH^high^CD44^high^ and ALDH^low^CD44^low^ cells from control and cisplatin-treated UM-SCC-22B (**A**) Histogram plot of log2 intensity values for 16 microarrays following batch adjustment. (**B**) Principal component analysis of batch adjusted 16 microarrays showing the 1st and 2nd components with the most variance. Grey circles–Control ALDH^high^CD44^high^; yellow squares–Control ALDH^low^CD44^low^; black diamonds–Cisplatin ALDH^high^CD44^high^; blue triangles–Cisplatin ALDH^low^CD44^low^. Exp #1: 1–4; Exp #2: 5–8; Exp #3: 9–12; Pilot Exp: 13–16. (**C**) Unsupervised hierarchical clustering with complete linkage and Euclidean distance was performed on only statistically significant probes (adjusted *p*-value < 0.05).

The pilot and larger microarray samples were processed with the same transcriptome kit and hybridized to the same microarray platform. We used bioinformatic analysis with R and Bioconductor packages to determine if the pilot and larger microarray experiments could be combined for downstream differential gene expression analysis. We utilized histogram and principal component analyses, which showed the pilot microarray samples exhibited the largest variance (Supplementary Figure 4C). To adjust for experiment batch differences, we used Combat [22], which utilizes empirical Bayes methods. After adjusting for the four experimental batches, both histogram and principal component analysis showed the four cell populations (Control ALDH^high^CD44^high^, Control ALDH^low^CD44^low^, Cisplatin ALDH^high^CD44^high^, Cisplatin ALDH^low^CD44^low^) were grouped closer together (Figure 3A, 3B; Supplementary Figure 4D).

Differentially expressed genes were determined using univariate comparisons (*e.g.* Cisplatin ALDH^high^CD44^high^ *versus* Control ALDH^high^CD44^high^) and adjusted for multiple testing comparisons. Genes with an adjusted *p*-value < 0.05 were considered statistically significant. Since the Affymetrix Human Gene ST 2.1 microarrays also include microRNAs, lincRNAs, and non-annotated probes, we focused on probes annotated with Entrez ID and HUGO gene symbols. In general, there were about 200–1000 genes differentially expressed between the groups (Table 1). We combined the genes from the four-univariate comparisons and performed unsupervised hierarchical clustering (Figure 3C). As expected, the four samples within each cell population clustered together since the four experimental batches were adjusted and we only considered differentially expressed genes.

**Table 1:** Number of differentially expressed genes for different comparisons

Group 1	Group 2	Total genes	Higher genes	Lower genes
Cisplatin ALDH^high^CD44^high^	Control ALDH^high^CD44^high^	231	115	116
Control ALDH^high^CD44^high^	Control ALDH^low^CD44^low^	363	81	282
Cisplatin ALDH^high^CD44^high^	Cisplatin ALDH^low^CD44^low^	605	104	501
Cisplatin ALDH^low^CD44^low^	Control ALDH^low^CD44^low^	1036	582	454

For each comparison, the total number of genes with an FDR adjusted *p*-value < 0.05, the number of genes higher in group 1, and the number of genes lower in group 1. Only genes with HUGO gene symbol are consider in this analysis.

**Figure 4:**
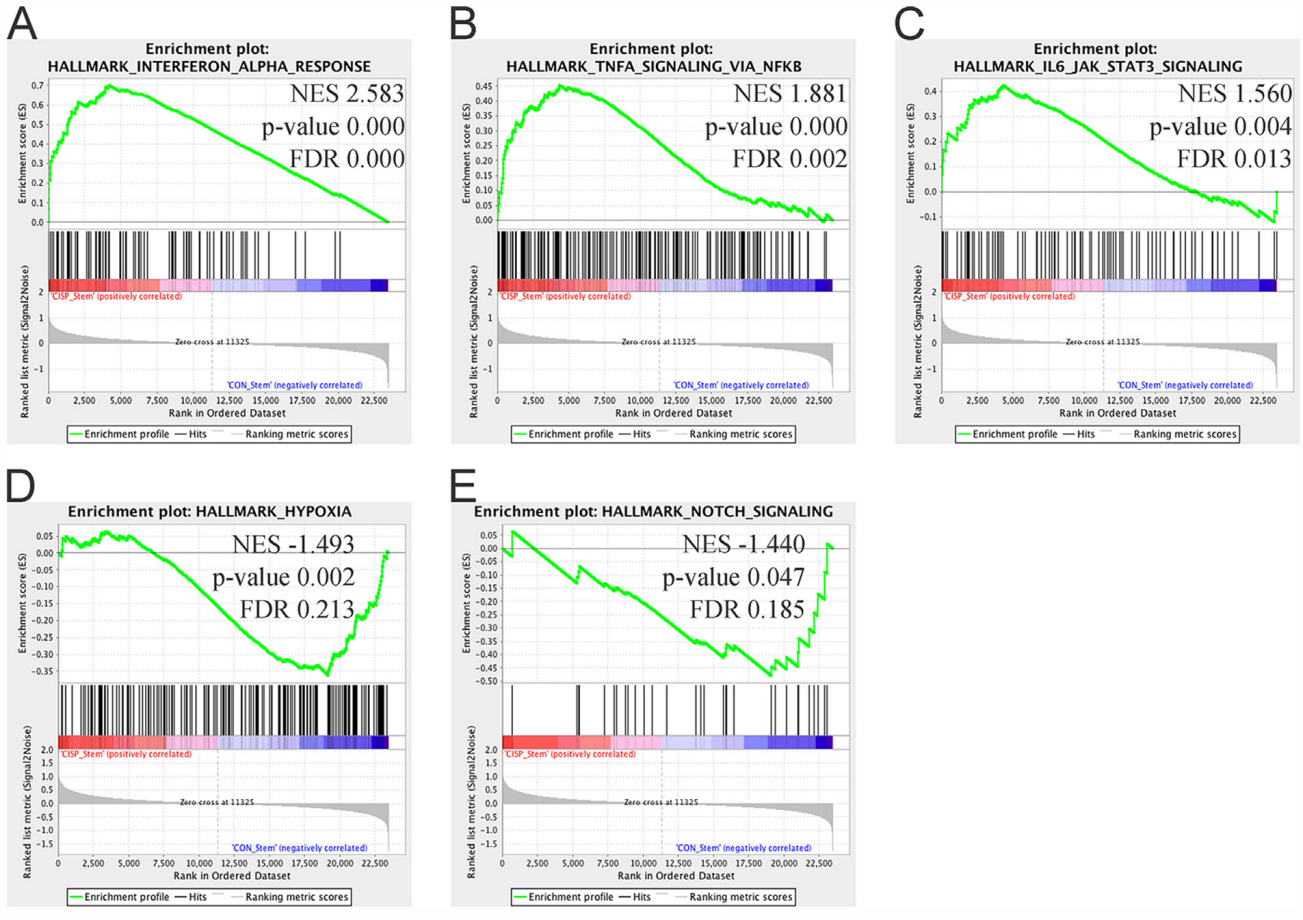
Gene Set Enrichment Analysis of cisplatin ALD^Hhigh^CD44^high^ vs. control ALDH^high^CD44^high^ cells Gene set enrichment analysis using “Hallmark” gene sets based on comparison between cisplatin ALDH^high^CD44^high^ and control ALDH^high^CD44^high^ cells. (**A**–**C**) Gene sets enriched in cisplatin ALDH^high^CD44^high^ cells with FDR < 0.25 and *p*-value < 0.05. (A) Interferon Alpha Response, (B) TNFα Signaling Via NF-κB, (C) IL6-JAK-STAT3 Signaling. (**D**–**E**) Gene sets enriched in control ALDH^high^CD44^high^ cells with FDR < 0.25 and *p*-value < 0.05. (D) Hypoxia, (E) Notch Signaling. NES–Normalized Enrichment Score.

To investigate the pathways modulated in cisplatin ALDH^high^CD44^high^ vs. control ALDH^high^CD44^high^, we used Gene Set Enrichment Analysis (GSEA) [23]. Recently, a new set of 50 gene sets, termed ‘Hallmarks’, was developed. These gene sets represent specific well-defined biological states or processes and display coherent expression. The Hallmark gene sets were generated by a computational methodology based on identifying gene set overlaps and extracting coherent representatives of them. We used GSEA with the Hallmark gene sets to evaluate cisplatin ALDH^high^CD44^high^ vs. control ALDH^high^CD44^high^. Among the highly statistically significant gene sets enriched in cisplatin ALDH^high^CD44^high^ were inflammatory signaling pathways, such as Interferon (IFN) Alpha Response (Genes up-regulated in response to IFNα proteins), TNFα Signaling Via NF-κB (Genes regulated by NF-κB in response to TNF), and IL6-JAK-STAT3 signaling (Genes up-regulated by IL6 via STAT3 during acute phase response) (Figure 4A–4C). Leading edge analysis of those three Hallmark pathways identified numerous genes in common, such as CXCL10, CXCL11, and IRF1 (Supplementary Table 2). Of note, only BST2, ICAM1, IL1β, IFIT2, and IFIH1 were statistically different based on univariate analysis with FDR adjustment. Only two Hallmark gene sets were statistically enriched in control ALDH^high^CD44^high^ vs. cisplatin ALDH^high^CD44^high^, including Hypoxia (Genes up-regulated in response to low oxygen levels (hypoxia)) and Notch Signaling (Genes up-regulated by activation of Notch signaling) (Figure 4D–4E).

While the GSEA results suggested the role of IFNα, TNFα, and/or IL6 signaling in regulating chemo-resistant ALDH^high^CD44^high^ cells, these secreted cytokines may be downstream effectors. We used a complimentary pathway analysis tool, iPathway, which is focused on KEGG pathways. The iPathway software analysis tool implements an ‘Impact Analysis’ approach that takes into consideration the direction and type of all signals on a pathway, the position, role and type of every gene, etc. [24, 25]. The Impact Analysis develops two *p*-values using two orthogonal approaches based on over-representation and the accumulated perturbation. These two *p*-values are combined into a global *p*-value for each pathway and iPathway adjusts for multiple testing based on Bonferroni and FDR approaches [24, 25]. Based on comparing only statistically significant genes between cisplatin ALDH^high^CD44^high^ vs. control ALDH^high^CD44^high^, we identified about 20 KEGG pathways with an unadjusted *p*-value < 0.05 (Supplementary Table 3). Adjusting for multiple testing reduced the number of significant pathways to 4–5, including Influenza A, Herpes simplex infection, Measles, Malaria, and Hepatitis C. These pathways all involve the response of the immune system. In addition, the TNF signaling and NF-κB signaling pathways were also enriched in the iPathway analysis. For the NF-κB signaling pathway, few genes expressed higher (*e.g.* DDX58, IL1β, ICAM1) in the cisplatin ALDH^high^CD44^high^ group compared to control ALDH^high^CD44^high^ (Supplementary Figure 5). Similarly, there was a mix of upregulated genes (IL1β, CCL20, ICAM1) and downregulated genes (MAPK10, MAP2K6) in the TNF signaling pathway (Supplementary Figure 6). Together, the GSEA and iPathway analyses identified similar immune system pathways in cisplatin-resistant ALDH^high^CD44^high^ cells.

Even though GSEA and iPathway analyses identified similar immune signaling pathways, there remained the possibility that these pathways were more downstream effectors. Furthermore, there is substantial crosstalk between the TNF, NF-κB, and IL6 signaling pathways hindering a more targeted approach to eradicating cisplatin ALDH^high^CD44^high^ cells. Therefore, we examined the 115 genes expressed higher in cisplatin ALDH^high^CD44^high^ cells compared to control ALDH^high^CD44^high^ cells (Table 1). As expected, there were numerous immune system related genes, but there were two secreted growth factors not related to the immune system, *e.g.* FGF2 and EREG (Supplementary Table 4). Examination of the 116 genes expressed lower in cisplatin ALDH^high^CD44^high^ cells did not identify obvious regulators of cell survival, proliferation, or apoptosis (Supplementary Table 5).

EREG (epiregulin) is a member of the EGF ligand family [26]. Various members of the EGF ligand family are secreted by HNSCC cells [27]. EREG is known to promote the proliferation of dental stem cells via the MAPK and JNK signaling pathways [28]. Furthermore, EGF ligand has been shown to regulate ALDH^high^CD44^high^ cells in HNSCC lines [29]. FGF2 is a well-known mitogen of fibroblasts and cancer cells. More recently, FGF2 has been shown to be required for maintaining human embryonic stem cells via promotion of self-renewal [30]. Marshall *et al.* showed FGFR signaling was dominant or co-dominant with EGFR in six HNSCC lines, whereas three lines exhibited little or no role for FGFRs and were highly EGFR dependent [31]. Therefore, HNSCC cell lines can be divided into subsets defined by sensitivity to EGFR and FGFR-specific inhibitors, which suggest FGFR inhibitors may represent novel therapeutics alone or in combination with EGFR inhibitors. Together, these data suggested FGF2 and/or EREG might regulate the resistance of ALDH^high^CD44^high^ cells to cisplatin.

**Figure 5:**
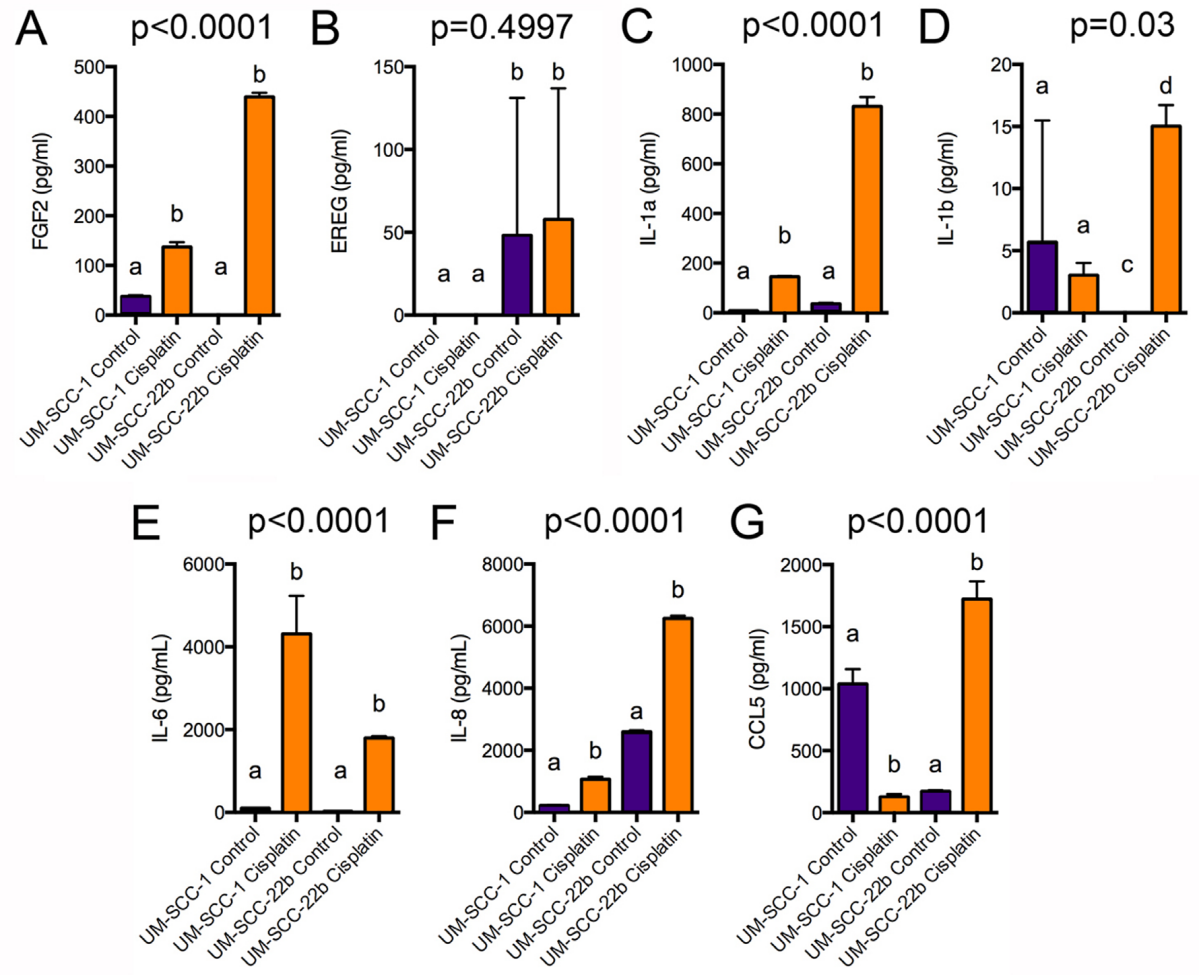
ELISA analysis of FGF2, EREG, and cytokine secretion UM-SCC-1 and UM-SCC-22B cells were treated for 5 days with 2 μM cisplatin, which was replaced every 2 days. Cell supernatant was centrifuged and analyzed by ELISA for (**A**) FGF2, (**B**) EREG, (**C**) IL-1α, (**D**) IL-1β, (**E**) IL-6, (**F**) IL-8, or (**G**) CCL5 secretion. Different letters depict statistically significant differences based on pairwise comparisons of control to cisplatin (*P <* 0.05).

We hypothesized that increased FGF2 and/or EREG secretion by cisplatin ALDH^high^CD44^high^ cells may contribute to the survival of these cells. Since FGF2 and EREG mRNA were increased in both cisplatin ALDH^high^CD44^high^ vs. control ALDH^high^CD44^high^ and cisplatin ALDH^low^CD44^low^ vs. control ALDH^low^CD44^low^ (Supplementary Figure 7), we anticipated that FGF2 and/or EREG would be secreted by both ALDH^high^CD44^high^ and ALDH^low^CD44^low^ cells following cisplatin treatment. We treated UM-SCC-1 and UM-SCC-22B cells for 5 days with 2 μM cisplatin and used ELISA to assay FGF2 and EREG protein in the media supernatant. As shown in Figure 5A, FGF2 secretion was dramatically increased in UM-SCC-1 and UM-SCC-22B cells following 5-day cisplatin treatment. Of note, FGF2 was expressed at low levels in untreated UM-SCC-1 cells, but not expressed in untreated UM-SCC-22B. However, EREG secretion was inconsistent between the triplicate wells and very low (Figure 5B).

The GSEA and iPathway analyses suggested a role for major signaling pathways in cisplatin-resistant ALDH^high^CD44^high^ cells (*e.g.* TNFα, IFN, IL6, NF-κB) that are known to regulate cell proliferation, survival, and participate in major biological processes (*e.g.* immune responses). However, IL1β (interleukin 1β) mRNA was the only cytokine statistically higher in cisplatin ALDH^high^CD44^high^ vs. control ALDH^low^CD44^low^. It was recently shown that exogenous IL6 treatment cooperates with cisplatin to further increase ALDH^high^CD44^high^ cells in HNSCC lines [21]. Therefore, we tested by ELISA if UM-SCC-1 and UM-SCC-22B secreted various cytokines in response to 5-day cisplatin treatment. In agreement with an increase with IL1β mRNA in cisplatin ALDH^high^CD44^high^ cells, there was an increase in IL1β secretion in UM-SCC-22B cells (Figure 5D). However, the levels of secretion were generally low and not statistically different in UM-SCC-1. IL1α, IL6, and IL8 secretion was highly induced in both UM-SCC-1 and UM-SCC-22B (Figure 5C, 5E, 5F). Interestingly, CCL5/Rantes secretion was reduced in UM-SCC-1 but increased in UM-SCC-22B (Figure 5G). Together, these data suggest that several major signaling pathways may regulate the pathobiology of cisplatin-resistant ALDH^high^CD44^high^ cells.

The dramatic increase in FGF2 secretion in both UM-SCC-1 and UM-SCC-22B cells following cisplatin treatment suggested cisplatin-resistant ALDH^high^CD44^high^ cells might utilize FGF signaling for survival. To inhibit the FGF2 signaling, we focused on testing an FGFR inhibitor. Recently, FGFR small-molecule kinase inhibitors with increased specificity compared to similar kinases (*e.g.* ABL, FYN, KIT, LCK, LYN, YES) have been reported. One such inhibitor, BGJ398, targets FGFR1, FGFR2, FGFR3, and FGFR4, and at nanomolar concentrations inhibits cell proliferation of BaF3 cells over expressing those receptors [32]. This inhibitor is currently in Phase II clinical trials for solid cancers, especially in patients with FGFR genetic alterations [33]. At nanomolar levels BGJ398 kills bladder cancer cells overexpressing wild-type FGFR3 (*e.g.* RT112, RT4, SW780, and JMSU1), but requires more than 3 μM to kill cells lacking this receptor [32]. Guagnano and collaborators tested 18 HNSCC cell lines and found that more than 8 μM BGJ398 was needed to kill these cells [33].

Here, we determined the BGJ398 IC_50_ for UM-SCC-1 and UM-SCC-22B cells. Both cell lines were seeded in 96-well plates for 3 or 5 days and treated at increasing doses of BGJ398. After 3 days of treatment, both UM-SCC-1 and UM-SCC-22B were killed with a BGJ398 IC_50_ concentration around 2.2 μM (Supplementary Figure 8A, Supplementary Table 6). However, after 5 day BGJ398 treatment, UM-SCC-22B required a higher IC_50_ concentration at 3.57 ± 0.26 μM (Supplementary Figure 8B, Supplementary Table 6). After 5 days, UM-SCC-1 cells were killed with an IC_50_ similar to 3-day treatment (1.91 ± 0.31 μM).

**Figure 6:**
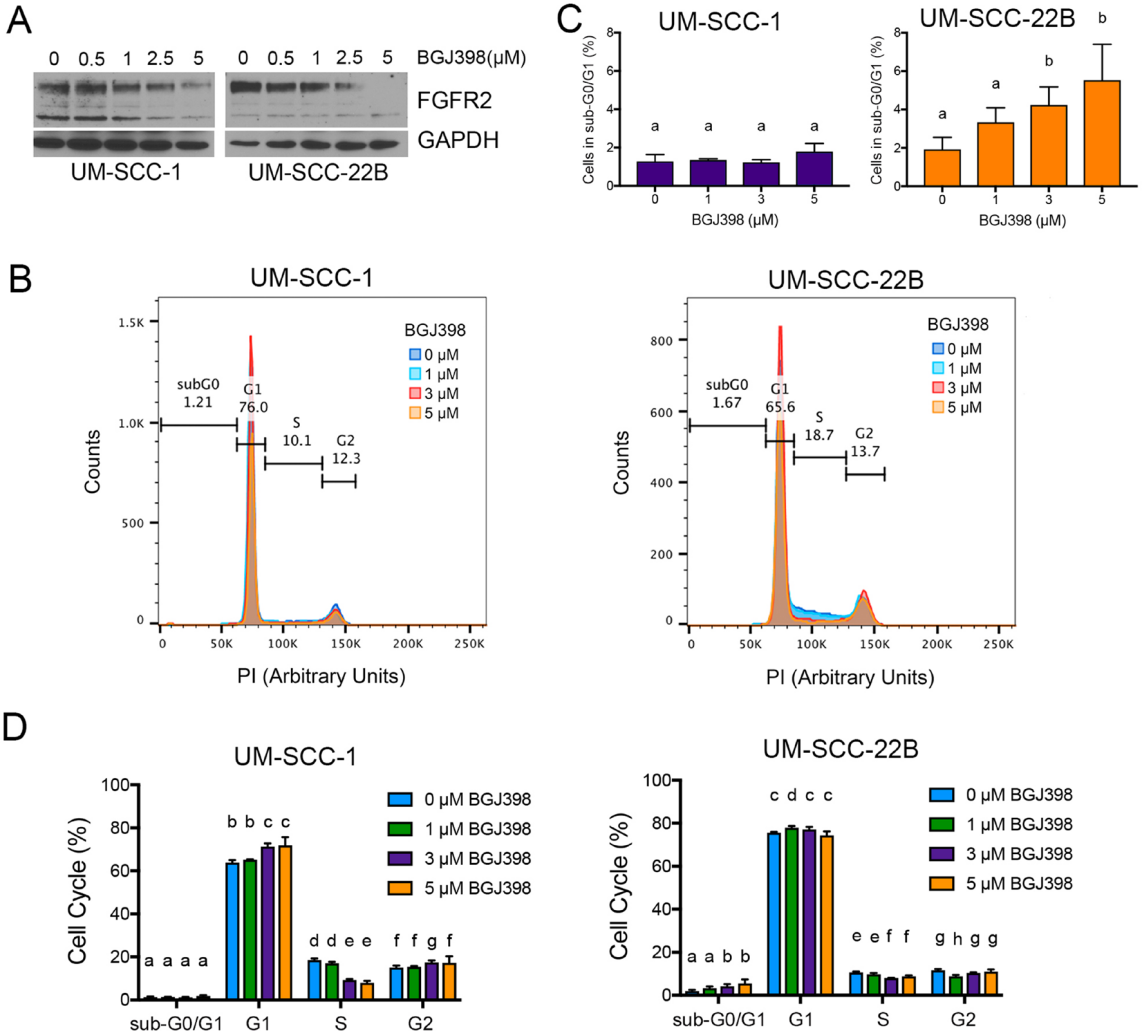
Effect of FGFR2 inhibition with BGJ398 on head and neck cancer cells (**A**) Western blot of UM-SCC-1 and UM-SCC-22B cells treated for 24 hours with increasing doses of BGJ398 (0.5–5 μM). (**B**–**D**) Cell cycle analysis with propidium iodide of UM-SCC-1 and UM-SCC-22B cells treated with increasing doses of BGJ398 (1–5 μM) for 24 hours. (B) Overlay of propidium iodide spectra for all conditions. (C) Overview of cells in sub-G_0_/G_1_. (D) Quantification of cells in each cell cycle stage. Different letters depict statistically significant differences based on multiple comparisons analysis. (*P <* 0.05).

**Figure 7:**
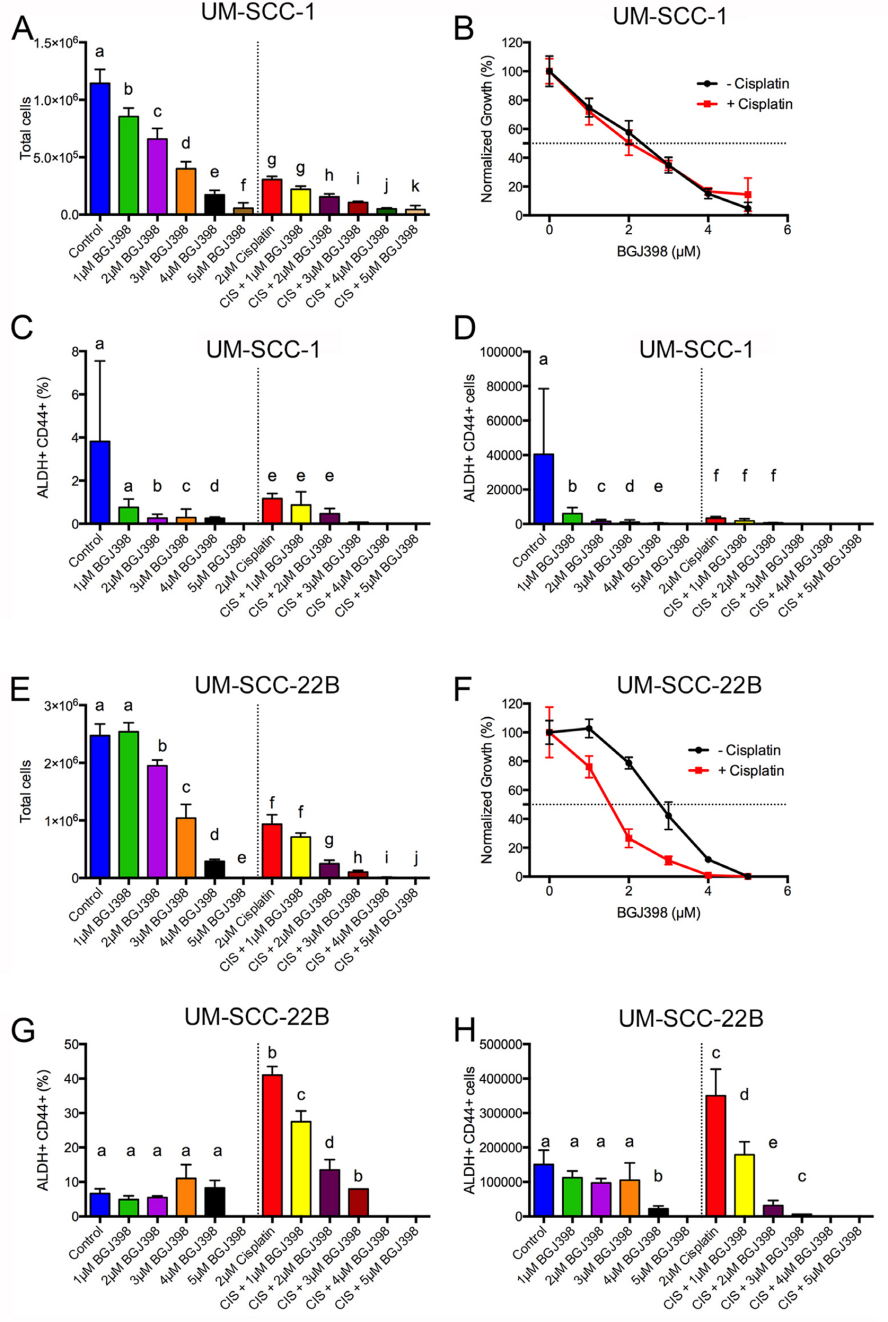
BGJ398 targeting of cisplatin-resistant ALDH^high^CD44^high^ cells 50,000 (**A**–**D**) UM-SCC-1 or (**E**–**H**) UM-SCC-22B cells were treated for 5 days with or without 2 μM cisplatin at various doses of BGJ398 (1-5 μM). Cells were harvested, counted, and stained with Aldefluor assay and CD44. (A, E) Number of total cells. (B, F) Normalized growth for total cells in the absence (black circles) or presence of 2 μM cisplatin (red squares). (C, G) Percentage of ALDH^high^CD44^high^ cells. (D, H) Number of ALDH^high^CD44^high^ cells. Average ± standard deviation. Different letters depict statistically significant differences based on pairwise comparisons to control (blue) or to cisplatin (red) (*P* < 0.05).

To verify the effect of BGJ398 in HNSCC cell lines, we treated UM-SCC-1 and UM-SCC-22B cells with increasing concentrations of BGJ398 for 24 hours. Western blots revealed a dose-dependent decrease in FGFR2 protein levels, thus confirming the ability of BGJ398 to inhibit the FGF signaling pathway (Figure 6A). To begin to understand the effect of therapeutic inhibition of FGFR2 in HNSCC, we performed propidium iodide (PI) staining of cells treated with increasing doses of BGJ398 (Figure 6B–6D). We observed that BGJ398 mediated a dose-dependent increase in apoptotic cells (sub-G_0_/G_1_ fraction) in UM-SCC-22B cells. On the other hand, BGJ398 caused a decrease in the fraction of cells in the S phase of cell cycle (without significant effect on apoptosis) in UM-SCC-1 cells, indicating that the effect of BGJ398 in this cell line was primarily to slow down in cell proliferation. While the mode of action of BGJ398 in these 2 cell lines was different, the net result in both is a decrease in the overall number of cells (Figure 7A, 7E).

To investigate if BGJ398 was able to target cisplatin-resistant ALDH^high^CD44^high^ cells, we tested BGJ398 treatment at various doses (1–5 μM) with or without 2 μM cisplatin. BGJ398 doses were chosen to flank the IC_50_ concentration at doses that had minimal toxicity or overt toxicity. UM-SCC-1 and UM-SCC-22B cells were treated in 6-well plates and treatments replaced every two days for a total of ~120 hr treatment. We examined the absolute number of cells, the frequency of ALDH^high^CD44^high^, and absolute number of ALDH^high^CD44^high^ cells. In agreement with the Alamar Blue data (Supplementary Figure 8, Supplementary Table 6), UM-SCC-1 cells were killed with a BGJ398 IC_50_ around 2.35 μM both in the presence or absence of 2 μM cisplatin (Figure 7A, 7B). The addition of 2 μM cisplatin dramatically reduced the number of UM-SCC-1 cells in combination with 3, 4, or 5 μM BGJ398. The massive cell loss at these doses prevented flow cytometry examination of ALDH^high^CD44^high^ frequency. Examination of ALDH^high^CD44^high^ frequency showed a statistical reduction at 2, 3, and 4 μM BGJ398 compared to the control group (Figure 7C). Unexpectedly, there was not an enrichment of ALDH^high^CD44^high^ cells at 2 μM cisplatin in this experiment. Furthermore, there was no statistically significant reduction of ALDH^high^CD44^high^ frequency for cisplatin + 1 μM BGJ398 or cisplatin + 2 μM BGJ398 when compared to cisplatin (Figure 7C). Given the sizable reduction in overall cell numbers (Figure 7A) and reduction in ALDH^high^CD44^high^ frequency (Figure 7C), it was expected that the overall number of ALDH^high^CD44^high^ cells decreased for most groups (Figure 7D).

We performed identical experiments with UM-SCC-22B cells to determine if BGJ398 could target cisplatin-resistant ALDH^high^CD44^high^ cells. In the absence of cisplatin, BGJ398 killed UM-SCC-22B cells with an IC_50_ of 2.91 ± 0.16 μM (Figure 7E, 7F). This result was similar to the Alamar Blue data (Supplementary Figure 8, Supplementary Table 6). In the presence of 2 μM cisplatin, the BGJ398 IC_50_ decreased to 1.49 ± 0.12 μM suggesting some cell-killing synergy between cisplatin and BGJ398. Due to the lack of cells at 5 μM BGJ398, cisplatin + 4 μM BGJ398, and cisplatin + 5 μM BGJ398 (Figure 7E), we were not able to examine ALDH^high^CD44^high^ cells. Additionally, there were only enough cells in the cisplatin + 3 μM BGJ398 group to examine ALDH^high^CD44^high^ without replicates, which prevented statistical testing. In contrast to the reduction of ALDH^high^CD44^high^ UM-SCC-1 cells by BGJ398 treatment (Figure 7C), BGJ398 as a single agent did not reduce ALDH^high^CD44^high^ UM-SCC-22B cells (Figure 7G). As expected, 2 μM cisplatin dramatically increased the ALDH^high^CD44^high^ percentage and overall cell number control compared to the control group (Figure 7G, 7H). When treated with 2 μM cisplatin there was substantial reduction in the percentage and absolute number of ALDH^high^CD44^high^ cells with increasing BGJ398 concentration. While there was a 25% reduction in overall cell number from cisplatin compared to cisplatin + 1 μM BGJ398, there was a 50% reduction in absolute ALDH^high^CD44^high^ cells suggesting the combination preferentially targets ALDH^high^CD44^high^ cells.

**Figure 8:**
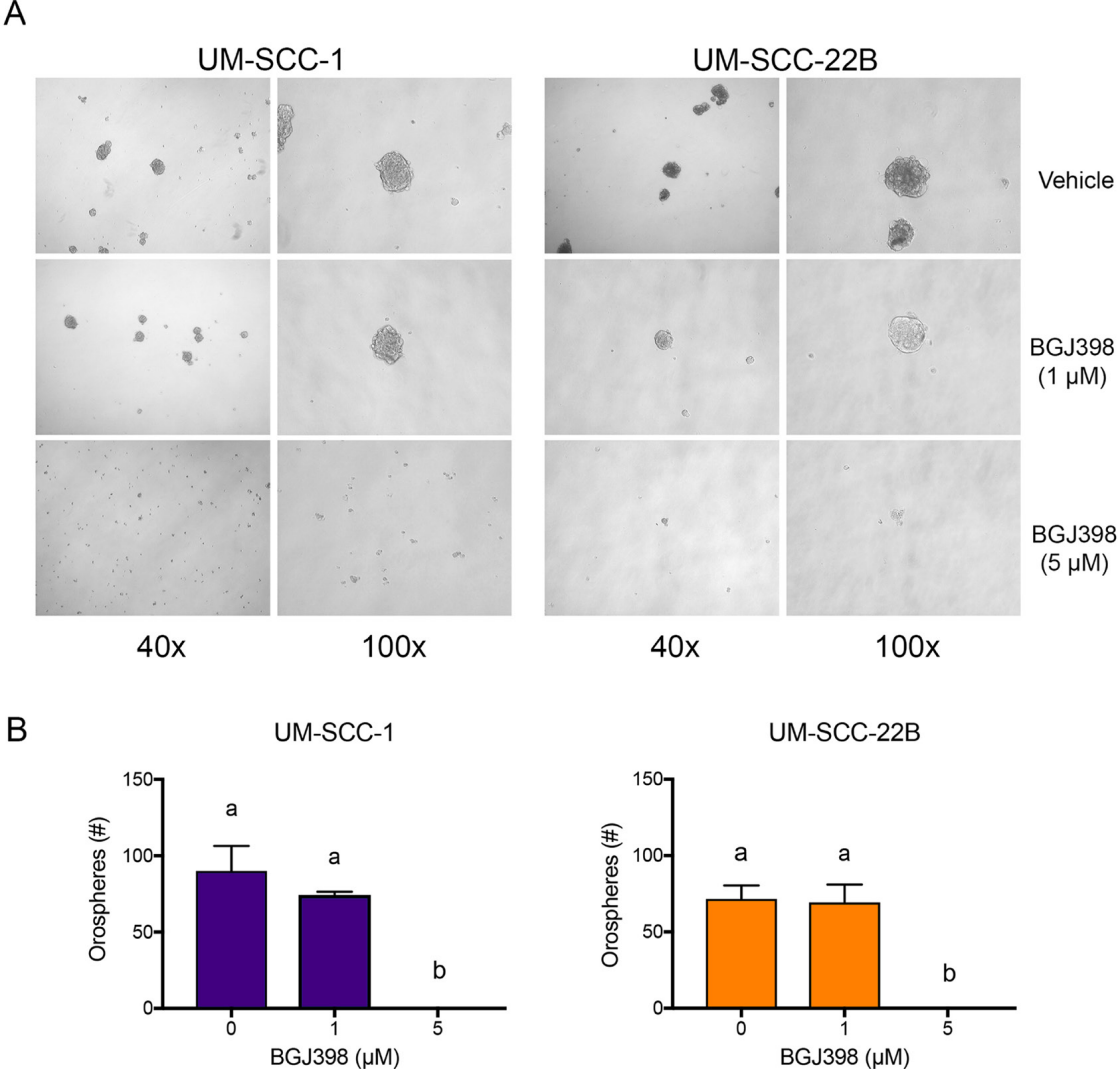
BGJ398 inhibits sphere formation of the UM-SCC-1 and UM-SCC-22B cells 12,000 UM-SCC-1 and UM-SCC-22B cells grown in ultralow attachment and serum-free conditions were treated with increasing doses of BGJ398. Spheres were counted 4 days post-treatment. (**A**) Representative micrographs of each condition taken at 40× and 100× magnification. (**B**) Quantification of formed spheres. Averages ± standard deviations. (*P* < 0.05).

We used the orosphere assay (*i.e.* survival/growth of head and neck tumor spheres in serum-free, ultra-low attachment conditions) to verify the impact of FGFR signaling on the phenotype of ALDH^high^CD44^high^ cells. Cells were plated at single-cell densities and were treated with increasing doses of BGJ398 for 4 days. Treatment with BGJ398 resulted in a dose-dependent decrease in the number of orospheres (Figure 8), confirming a role for FGFR signaling in *in vitro* stemness properties of head and neck cancer cells.

**Figure 9:**
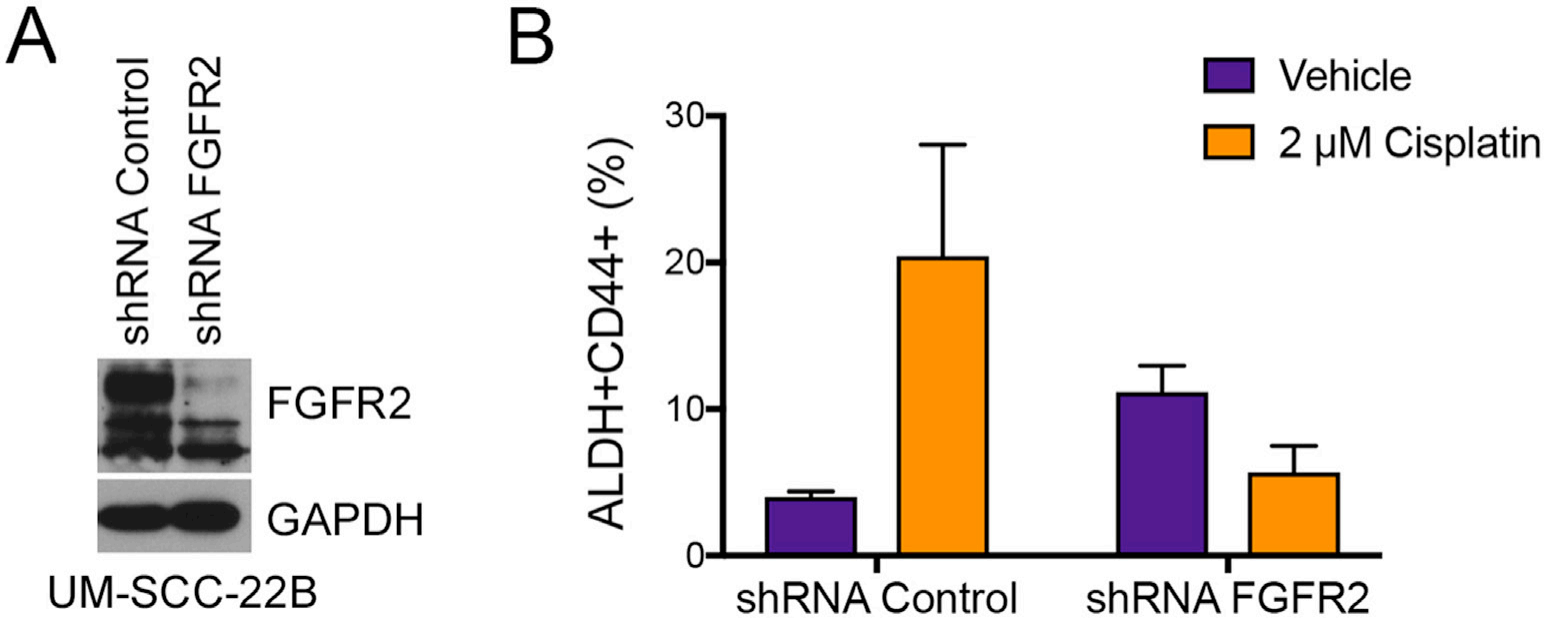
FGFR2 mediates cisplatin-induced increase in the fraction head and neck cancer stem cells Stable knockdown of FGFR2 was achieved using lentiviral shRNA constructs. (**A**) Western blot demonstrating successful shRNA-mediated knockdown of FGFR2 in UM-SCC-22B cells. (**B**) Analysis of the fraction of cancer stem cells (i.e. ALDH^high^CD44^high^) cells after 4 days of treatment with 2 μM Cisplatin. Averages ± standard deviations.

To verify the results obtained with BGJ398, we silenced FGFR2 in UM-SCC-22B cells using stably transduced shRNA constructs encoded in lentiviral vectors. We first confirmed silencing of FGFR2 via western blot analysis (Figure 9A). We then examined the impact of FGFR2 in the fraction of ALDH^high^CD44^high^ cells upon treatment with cisplatin. As expected, cisplatin treatment of the shRNA-control cells (scrambled sequence) mediated an increase in the fraction of cancer stem cells (ALDH^high^CD44^high^). In contrast, cisplatin no longer increased the fraction ALDH^high^CD44^high^ cells in FGFR2-silenced cells (Figure 9B), mimicking results obtained with the FGFR inhibitor BGJ398 (Figure 7G).

## DISCUSSION

The relative resistance of ALDH^high^CD44^high^ HNCSCs to chemotherapy represents a therapeutic challenge. These cells are predicted to mediate recurrence and metastatic spread, which ultimately leads to organ failure and eventual death from HNSCC. Current treatments for HNSCC include chemotherapy and radiation therapy for the vast majority of patients. Except for cetuximab, the EGFR monoclonal antibody, there are no molecularly targeted therapeutics for HNSCC. The addition of cetuximab to chemotherapy prolonged the median progression-free survival time from 3.3 to 5.6 months and overall survival from 7.4 to 10.1 months [10]. Therapeutic targeting of chemo-resistant HNCSCs requires a greater understanding of the molecular pathways within these cells.

We met this need with microarray analysis of cisplatin-resistant ALDH^high^CD44^high^ cells and therapeutic targeting of those cells by inhibition of the FGF pathway. Cisplatin represents standard chemotherapeutic treatment for HNSCC and we utilized published cisplatin IC_50_ concentrations (2 μM) for two HNSCC cell lines. At 2 μM cisplatin, there was a significant and consistent increase in ALDH^high^CD44^high^ cells around 5 days of treatment (Figure 1). A pilot microarray of control and cisplatin-treated ALDH^high^CD44^high^ and ALDH^low^CD44^low^ cells identified a few upregulated genes in cisplatin-resistant ALDH^high^CD44^high^ cells compared to control ALDH^high^CD44^high^ cells, including FGF2, EREG, AREG, and SPRR1B. SPRR1B was previously reported to be expressed in HNSCC ALDH^high^ cells [36]. The EGF family is known to regulate HNSCC growth, EGFR kinase inhibitors are currently in clinical development for HNSCC treatment, and cetuximab is approved for the treatment of HNSCC [6, 10, 29]. There were 235 probesets higher or lower in cisplatin-resistant ALDH^high^CD44^high^ cells, suggesting that additional microarray experiments would provide a robust set of genes for downstream pathway and investigational analysis.

Based on these initial microarray results, we performed additional microarrays of control and cisplatin ALDH^high^CD44^high^ and ALDH^low^CD44^low^ cells. To increase statistical power, we combined the microarray data from the pilot and subsequent experiments. However, there were obvious batch effects, particularly for the samples analyzed in our pilot studies (Supplementary Figure 4), which affected the discovery of differentially expressed genes. Following adjustment of batches and experiments using ComBat [22], we performed pairwise comparisons to identify differentially expressed genes. Bioinformatic analysis using two complementary approaches, GSEA [25] and iPathway [24, 25], were used to identify potential pathways regulating cisplatin-resistant ALDH^high^CD44^high^ cells. Both pathway analyses identified an enrichment of immune signaling pathways, including TNFα, IFN, IL6-JAK-STAT3, and NF-κB.

There is growing literature showing a role for “traditional” immune signaling pathways in regulating cancer and CSCs. IL6/STAT signaling is known to increase breast CSCs [35, 36] and HNCSCs [18, 21]. Tocilizumab, an IL6 receptor monoclonal antibody, is FDA-approved for rheumatoid arthritis and pre-clinical studies have shown tocilizumab targets CSCs [18, 36]. In addition, STAT inhibitors are known to target CSCs [37]. In contrast to IL6/STAT signaling, very little data exists showing a direct role of TNF signaling on CSCs. Recently, Zhang *et al.* showed that exogenous TNF increased expression of Oct4, Nanog, and BMI1, genes related to “stemness”, in renal cell carcinoma cells [37]. TNF also increased tumorsphere formation by renal cell carcinoma cells and induced an epithelial-mesenchymal transition. Both tumorsphere formation and epithelial-mesenchymal transition are associated with stemness [38–40]. The NF-κB pathway is also known to regulate CSCs, especially in breast cancer, and NF-κB inhibitors target CSCs [41–43].

In agreement with the pathway analysis of the microarray data, we detected significant secretion of IL1α, IL6, and IL8 in both UM-SCC-1 and UM-SCC-22B cells treated with cisplatin (Figure 5). IL1β secretion was induced by cisplatin in only UM-SCC-22B cells. Interestingly, CCL5 secretion was decreased by cisplatin in UM-SCC-1 but increased in UM-SCC-22B cells. CCL5 was found, in combination with FGF2, phospholipase C (PLCg2), frizzled receptor-4 (FZD4), and chemokine [C-X3-C motif] (CX3CL1), to be overexpressed in bevacizumab-resistant HNSCC [44]. Antiangiogenic agents, such as bevacizumab, increase breast CSCs via tumor hypoxia [45], which might also be via up regulation of CCL5. It has been reported that HNSCC has been shown to express a number of chemokines, and their receptors, which may promote chemotherapy resistance [46]. At the mRNA level, most HNSCC lines express CCL5, CCL20, CXCL1, CXCl2, CXCL3, CXCL10, and CXCL11 [47]. Here, we found CCL20 was expressed 2-fold higher in cisplatin-resistant ALDH^high^CD44^high^ cells compared to control ALDH^high^CD44^high^ cells suggesting future studies of CCL20 may provide insight into chemotherapy resistance.

The microarray data suggest a substantial induction of immune signaling pathways by cisplatin-resistant cells. It is possible that increased cytokine and chemokine secretion by cisplatin-resistant ALDH^high^CD44^high^ and ALDH^low^CD44^low^ cells would attract immune cells within the context of a tumor. These immune cells would secrete additional cytokines and chemokines that could provide survival signals to ALDH^high^CD44^high^ cells. This bystander effect was demonstrated in breast CSCs resistant to chemotherapy by the secretion of IL8 by dying cancer cells that signaled survival signals via the IL8 receptor, CXCR1, on the CSCs [48]. While our experiments examined cisplatin-resistant ALDH^high^CD44^high^ cells *in vitro*, it would be interesting to perform similar microarray experiments with HNSCC tumor xenografts in mice. Based on the substantial utilization of immune signaling pathways by HNCSC and the secretion of numerous cytokines, it is likely that cisplatin-resistant HNCSC *in vivo* would be more reliant on these immune signaling pathways.

A major goal of this work was to identify mechanisms/pathways utilized by cisplatin-resistant ALDH^high^CD44^high^ cells and therapeutically target those resistance pathways. It was beyond the scope of this work to investigate each immune signaling pathway. Future studies could focus on the role of TNF and/or IFN signaling in cisplatin-resistant ALDH^high^CD44^high^ cells. There are FDA-approved therapies to block TNF, including Remicade (infliximab), Enbrel (etanercept), Humira (adalimumab), Cimzia (certolizumab pegol) and Simponi (golimumab). However, there are substantial risks with TNF inhibitors, including increased risk of development of solid cancers in patients with rheumatoid arthritis [49].

To focus efforts on rational therapeutic targeting of cisplatin-resistant ALDH^high^CD44^high^ cells, we investigated FGF2 and EREG. The EGF family is well known to regulate HNSCC. HNSCC can express EGFR and expression is associated with worse outcome [7]. Various members of the EGF ligand family are secreted by HNSCC cells [27]. EREG is known to promote the proliferation of dental stem cells via the MAPK and JNK signaling pathways [28]. However, we failed to detect significant secretion of EREG protein following cisplatin treatment. Due to limited ELISA reagents, the EREG experiment was performed once with triplicate wells. This negative result may be due to EREG secretion being below detection levels in the ELISA. Based on the microarray mRNA data, it appears that EREG is more preferentially expressed by the ALDH^high^CD44^high^ cells compared to the ALDH^low^CD44^low^, which constitute the majority of the cells in both UM-SCC-1 and UM-SCC-22B cell lines. Further experiments whereby ALDH^high^CD44^high^ and ALDH^low^CD44^low^ cells are FACS sorted and treated separately in small micro-well plates may uncover a role of EREG secretion in cisplatin ALDH^high^CD44^high^ cells.

Based on the substantial secretion of FGF2 following cisplatin treatment of UM-SCC-1 and UM-SCC-22B, we investigated whether inhibition of FGF signaling would target cisplatin-resistant ALDH^high^CD44^high^ cells. The role of FGF signaling in HNSCC is poorly studied, but Nguyen *et al.* showed FGFR1 was highly expressed in 54% of HNSCC cases and was significantly correlated with malignant behavior [50]. Treatment of HNSCC lines with the FGFR inhibitor PD173074 reduced cell proliferation at low nanomolar concentrations [50]. However, PD173074 is not currently being tested in active clinical trials, which limits potential future studies.

Amongst the commercially available potent and selective FGFR inhibitors that are in clinical trials, we selected BGJ398 to investigate if FGFR inhibition targeted cisplatin-resistant ALDH^high^CD44^high^ cells. BGJ398, targets FGFR1, FGFR2, FGFR3, and FGFR4 and at nanomolar concentrations inhibits cell proliferation of BaF3 cells over expressing those receptors [32]. This inhibitor is currently in Phase II clinical trials for solid cancers, especially in patients with FGFR genetic alterations [33]. Guagnano *et al.* tested 18 HNSCC cell lines and found that more than 8 μM BGJ398 was needed to kill these cells when tested in 1536-well plates [33]. This is in contrast to nanomolar inhibition of HNSCC cell proliferation by PD173074 [50].

Here, we determined the BGJ398 IC_50_ concentration for UM-SCC-1 and UM-SCC-22B to be between 2–3.5 μM depending on 3 or 5-day treatment. This is above the plasma C_max_ levels achieved in pre-clinical animal mouse models (0.86 μM–5 mg/kg IV; 0.42 μM–20 mg/kg oral gavage) or rat models (0.97 μM–5 mg/kg IV; 0.26 μM–10 mg/kg oral gavage) [34]. Based on this information, we tested a range of BGJ398 doses from 1 to 5 μM for the ability to target cisplatin-resistant ALDH^high^CD44^high^ cells. Surprisingly, BGJ398 was able to reduce UM-SCC-1 ALDH^high^CD44^high^ cells as a single agent at 2–4 μM, which might be due to the low basal FGF2 secretion by UM-SCC-1 cells. In UM-SCC-22B cells, BGJ398 dramatically reduced cisplatin-resistant ALDH^high^CD44^high^ cells at 1–2 μM, but had no effect as a single agent. This reduction at doses near the pre-clinical C_max_ suggests that BGJ398 might be able to target cisplatin-resistant ALDH^high^CD44^high^ cells in animal models. Furthermore, UM-SCC-22B was developed from a neck metastasis derived from a patient who had a primary tumor in the hypopharynx [51]. Since HNCSCs are predicted to mediate metastasis, the combination of cisplatin and BGJ398 might reduce the primary tumor burden and metastatic spread of UM-SCC-22B cells in animal models.

In conclusion, microarray analysis of HNCSCs from control and cisplatin-treated cells showed an enrichment of major signaling pathways, such as IFN, TNF, IL6/STAT, and NF-κB, in cisplatin-resistant ALDH^high^CD44^high^ cells. We demonstrated an increase in cytokine secretion by ELISA following cisplatin treatment. Microarray analysis showed FGF2 and EREG mRNA increased in cisplatin ALDH^high^CD44^high^ cells. We found a substantial increase in FGF2 secretion following cisplatin treatment, but no statistical increase in EREG secretion. Finally, treatment of HNSCC cells with the FGFR inhibitor BGJ398 and cisplatin was able to reduce ALDH^high^CD44^high^ cells in the UM-SCC-22B cell line at low micromolar levels. As a single agent, BGJ398 reduced ALDH^high^CD44^high^ cells in the UM-SCC-1 cell line. The *in vitro* work presented here was informed by *in vivo* experiments that demonstrated that cisplatin treatment increases the fraction of head and neck cancer stem cells [21]. In search for a mechanistic explanation for these findings, we unveiled a significant role for FGFR signaling in the development of cisplatin resistance. Collectively, these data suggest that patients with head and neck cancer might benefit from targeting of cisplatin-resistant ALDH^high^CD44^high^ cells by therapeutic inhibition of FGFR.

## MATERIALS AND METHODS

### Reagents

6-well (3516), 96-well tissue culture (3596), 96-well round bottom (3799), T75 (430641) flasks, and 35 um filter cap FACS tubes (352235) were from Corning. DMEM (11960), Penicillin-Streptomycin (15070, 5000 U/mL), Glutamax (100X, 35050-061), Sodium Pyruvate (11360, 100 mM), and 0.25% trypsin/1 mM EDTA (25200) were from Invitrogen. Fetal Bovine Serum (SH30396.03) was from Hyclone. AO/PI (F23001) and Luna (L12002) slides were from Logos Biosystems. CD44-APC (clone G44-26; #559942) was from BD Biosciences. DMSO (D2650), resazurin (R7017), and DAPI (D8417) were from Sigma. EREG ELISA (SEB945HU) was from Cedarlane. RNeasy Mini Kit (74106) was from Qiagen. RNA 6000 Pico and Nano Kits were from Agilent. Ovation Pico WTA System Kit was from NuGEN. Human Gene ST 2.1 microarrays were from Affymetrix. Clinical-grade cisplatin (Teva Pharmaceuticals; 1mg/mL) was obtained from University of Michigan Hospital Pharmacy. BGJ398 (S2183) was from Selleckchem and dissolved in DMSO to 1.78 mM. DEAB (01705) and Aldefluor Assay Buffer (01702) were from StemCell Technologies. Aldefluor reagent, BAAA-DA, was synthesized by the University of Michigan Vahlteich Medicinal Chemistry Core.

### Cell culture

UM-SCC-1 and UM-SCC-22B cells [51] (gift from Thomas Carey) were cultured in DMEM/10% FBS/1% PenStrep/3X Glutamax/1 mM Sodium Pyruvate and plated at 2,500 cells/cm^2^ in T75 flasks. Cells were passaged every 3–4 days and counted using the Luna FL cell counter with AO/PI dye.

### Alamar blue (resazurin) assay

Resazurin powder was dissolved in PBS at 8 mM and filtered. It was further diluted to 440 μM with PBS and filtered [52].

### IC_50_

1,000 UM-SCC-1 or 2,000 UM-SCC-22B cells [51] were seeded per well in 95 μL of media in 96-well tissue culture plates. Drugs were diluted with media and 5 μL transferred to 95 μL of cells/media. Cell growth, based on mitochondrial redox potential, was measured at various time points using the Alamar Blue (resazurin) assay. 10 μL (~10% vol/vol) of 440 μM resazurin was added to each well and incubated for 1–4 hrs at 37° C. Fluorescence, as a measure of cell growth, was measured with a BioTek Synergy plate reader by exciting at 530 nm and reading emission at 590 nm. Fluorescence was normalized to 0% with wells without media and to 100% with wells containing untreated cells. Data was normalized and curve fitted using Prism 6. Curves were fitted using non-linear regression based on the formula:

Y=Bottom+Top−Bottom1+(XEC50)−Hill coefficient

### ELISA

50,000 cells were seeded in 2 mL media in triplicate wells in 6-well plates (~5,000 cells/cm^2^). The following day (Day 1), media was replaced with fresh media containing 2 μM cisplatin. Media was replaced on days 3 and 5. On day 6, 1000 μL media was centrifuged at 2000 g for 5 min at 4° C to pellet cells. 200 μL of media was transferred to round-bottom 96-well plates. ELISAs were performed according to manufacturer instructions at the University of Michigan Cancer Center ELISA Core.

### Flow cytometry

50,000 UM-SCC-22B cells were plated in 12 wells in 2 mL media in two 6-well plates. The following day, media was replaced with fresh media with or without 2 μM cisplatin. Media was replaced on days 3 and 5. On day 6, cells were harvested with 0.25% trypsin, counted, filtered through 35 μm membranes into 5 mL FACS tubes, and centrifuged at 337 g for 5 min at 4C. Cell pellets were suspended in 985 μL Aldefluor Assay Buffer. 10 μL of 0.1 mg/mL DAPI was added. 5 μL of DEAB, as an inhibitor of the Aldefluor reaction, was added to a separate 5 mL FACS tube. 5 μL of activated Aldefluor reagent was added to 995 μL of cells/DAPI. 500 μL of this solution was immediately transferred to the tube containing DEAB. Cells were incubated for 30 min at 37° C. Tubes were centrifuged at 337 g for 5 min at 4° C and placed on ice. Cell pellets were suspended in 300 μL Aldefluor Assay Buffer. 0.5 μL CD44-APC was added to tubes, except the DEAB control tube. Samples were incubated for exactly 15 min on ice and then centrifuged. Cell pellets were suspended on ice in Aldefluor assay buffer for a final concentration of 1–3 × 10^6^ cells/mL. Flow cytometry sorting was performed on a Beckman Coulter MoFlo XDP with 355 nm, 488 nm, and 633 nm lasers with appropriate filters. Gating for CD44^high^ and ALDH^high^ events were determined using the DEAB sample as a fluorescence minus one control. Gates for CD44^high^ and ALDH^high^ cells were set at 0.1%. ALDH^high^CD44^high^ and ALDH^low^CD44^low^ cells from control and cisplatin groups were collected into separate 5 mL FACS tubes containing 1 mL of PBS/3% FBS. For flow cytometry analysis, samples were run on a Beckman Coulter CyAn ADP with 405 nm, 488 nm, and 633 nm lasers with appropriate filters. Data for at least 10,000 live cells were collected per each sample.

### RNA extraction, microarray, bioinformatic analysis

See Supplementary Materials and Methods for in depth description. The dataset (CEL files and ComBat intensity values) has been deposited in Gene Expression Omnibus (GEO; http://www.ncbi.nlm.nih.gov/geo/) of the National Center for Biotechnology Information with accession number GSE72384.

### Western blots

Whole cell lysates were prepared using NP-40 lysis buffer (1% Nonidet P-40, 50 mM Tris-HCL, pH 7.4, 10% glycerol, 2 mM MgCl, and 200 mM NaCl) containing protease inhibitors. UM-SCC-1 and UM-SCC-22B cells were treated with BJG389 at 0.5, 1, 2.5, or 5 μM concentrations for 24 hours. Proteins were resolved using 9% SDS-PAGE. Membranes were probed using antibodies at 1:500 dilution against human FGFR2 (Santa Cruz Biotechnology; Santa Cruz, CA, USA) and at 1:1,000,000 dilution against human GAPDH (Chemicon International, Millipore; Temecula, CA). Primary antibodies were incubated overnight at 4° C, while secondary antibodies were incubated for 2 hours at room temperature.

### Orospheres

Orospheres were cultured in DMEM/F-12 (Invitrogen) supplemented with 10 ng/ml EGF (Sigma-Millipore), 10ng/ml bFGF (Millipore), 1% penicillin/streptomycin (Invitrogen), 1% glutamax (Invitrogen), 1% N-2 supplement (Invitrogen), as we described [53]. Unsorted UM-SCC-1 or UM-SCC-22B cells were counted using a hemocytometer, diluted to 12,000 single cells per 1.5 ml of orosphere media, and added to 6-well ultra-low attachment plates (Corning; Corning, NY, USA). Cells were treated with vehicle or BJG389 (Selleckchem, USA) for 24 hours by adding 500 μL of orosphere media gently on top of the wells for a final volume of 2 mL. Colonies of 25 cells or more were considered orospheres and were counted after 4 days.

### Cell cycle analysis

For cell cycle analysis, 4 × 10^5^ UM-SCC-1 or UM-SCC-22B cells were plated in 6-well plates. The next day, cells were treated with either vehicle or BJG398 (Selleckchem, USA) for 24 hours. Both supernatant and adherent cells were harvested. Cells were then exposed to 0.1% sodium citrate, 0.1% Triton X-100, 50 μg/mL propidium iodide (Sigma-Aldrich), and 100 μg/mL RNaseA. Flow cytometry was conducted in the University of Michigan Flow Cytometry Core (LSRFortessa; BD Biosciences). The percentage of cells in each cell-cycle phase was analyzed using FlowJo software (FlowJo, LLC; Ashland, Oregon, USA). Data was averaged from triplicates.

### FGFR2 silencing

Second-generation shRNA lentiviral vectors and packaging plasmids were obtained from the University of Michigan Vector Core. HEK293T cells were used for lentiviral particle production. Cells were transiently co-transfected by the calcium phosphate method with lentiviral packaging vectors psPAX2, pMD2G and shRNA-FGFR2 or shRNA-scramble sequence control (shRNA-C). UM-SCC-1 and UM-SCC-22B cells were infected with the virus-containing supernatants in presence of 4 μg/mL polybrene and selected with 1μg/ml puromycin (Sigma-Aldrich, St. Louis, Missouri, USA) for 2 weeks.

### Statistical analysis

All experiments were performed in at least triplicate per group and repeated at least twice. Except for the bioinformatics analysis, statistical significance was determined in Prism 6 using one-way ANOVA with Sidak's or Tukey's adjustment for multiple comparisons. Adjusted *p*-values < 0.05 were considered significant.

## SUPPLEMENTARY MATERIALS FIGURES AND TABLES






