# Supplementary material for: FGFR signaling regulates resistance of head and neck cancer stem cells to cisplatin
Source: Oncotarget. 2018 May 18;9(38):25148–65. doi: 10.18632/oncotarget.25358 (PMC5982758; doi:10.18632/oncotarget.25358)
Supplement: Supplementary file 1 [file oncotarget-09-25148-s001.pdf]

FGFR signaling regulates resistance of head and neck cancer stem cells to cisplatin

SUPPLEMENTARY MATERIALS

RNA extraction

Collected cells from flow cytometry were centrifuged at 337 g for 5 min at 4C. Excess volume was removed with a p1000 pipette and the remaining sample transferred to 1.5 mL microcentrifuge tube. Tubes were centrifuged at 300 g for 5 min at 4C. Supernatant was removed with a p200 pipette until <30 µL sample remained. RNA was extracted using the Qiagen RNeasy Mini Kit following the manufacturer instructions. Briefly, 350 µL RLT was added to cell pellets (in <30 µL) and vortexed for 30 sec. 350 µL 70% EtOH was added to each sample and mixed until no viscosity remained. The sample was transferred to spin column, centrifuged 8000 g × 15 sec. Then 700 µL RW1 was added to spin column, centrifuged 8000 g × 15 sec. Then 500 µL RPE buffer to spin column, centrifuged 8000 g × 15 sec. Then 500 µL RPE buffer to spin column, centrifuged 8000 g × 2 min. Spin columns were placed on new collection tube and centrifuged 8000 g × 1 min. Spin columns were placed on 1.5 mL RNAse-free centrifuge tubes. 15 or 30 µL of RNAse-free water added to membrane and then centrifuged 8000 g × 1 min. 1 µL of sample was used to measure RNA concentration with a Nanodrop.

Microarray

Microarrays and RNA processing were performed by the University of Michigan Microarray Core facility. The quality of total RNA for each sample was confirmed with a Bioanalyzer with either a Eukaryote Total RNA Pico or Nano kit. RNA Integrity Numbers (RIN) for samples were from 7.8–10.00 indicating high quality RNA. Over 4 separate experiments, approximately 13.5 pg RNA/cell was recovered. At least 10ng of total RNA was processed with the Ovation Pico whole transcriptome amplification kit and hybridized with Human Gene ST 2.1 plate using manufacturer instructions.

Bioinformatic analysis

CEL files processed using R and Bioconductor after loading the following, and associated, packages: oligo [1], genefilter [2], affycoretools [3], limma [4], ReportingTools [5], sva [6], gplots [7], hwriter [8], pd.hugene.2.1.st [9], hugene21sttranscriptcluster.db [10]. Files were preprocessed by Robust Multiarray Average (RMA) with background substraction, quantile normalization, and median polish. Data was fitted with robust probe level linear models to all the probesets for quality control analysis. Since microarrays were performed on two different days and flow cytometry performed on four separate days, batch effects (experiments) were accounted for using ComBat [11] in the sva package. Boxplot and histogram analysis showed similar profiles for the 16 microarray samples. Principal component analysis (PCA) following experiment adjustment showed clustering of similar populations (i.e. control ALDH^{high}CD44^{high}). Differentially expressed genes were identified using univariate comparisons [12]. First, the relative reliability of each array is estimated by measuring how well the expression values for that array follow the linear model. Second, data is fitted to a linear model (limma package) [12]. Third, the estimated coefficients and standard errors are computed. Finally, initial statistics are determined using an empirical Bayesian model. Multiple testing comparisons were adjusted using Benjamini and Hochberg (aka FDR) [13]. Probes with an adjusted p-value < 0.05 were considered statistically significant. Unsupervised hierarchical clustering with complete linkage and Euclidean distance was performed on only statistically significant probes. The dataset (CEL files and ComBat intensity values) has been deposited in Gene Expression Omnibus (GEO; http://www.ncbi.nlm.nih.gov/geo/) of the National Center for Biotechnology Information with accession number GSE72384.

Pathway analysis

Gene Set Enrichment Analysis (GSEA) and iPathway analysis platforms were used. GSEA is a computational method that determines whether an a priori defined set of genes shows statistically significant, concordant differences between two biological states (e.g. phenotypes) [14]. The iPathway software analysis tool implements an ‘Impact Analysis’ approach that takes into consideration the direction and type of all signals on a pathway, the position, role and type of every gene, etc. [15, 16]. The Impact Analysis develops two p-values using two orthogonal approaches based on over-representation

and the accumulated perturbation. These two *p*-values are combined into a global *p*-value for each pathway and iPathway adjusts for multiple testing based on Bonferroni and FDR approaches [15, 16].

REFERENCES

1. Carvalho BS, Irizarry RA. A framework for oligonucleotide microarray preprocessing Bioinformatics. 2010; 26:2363–2367.
2. Gentleman R, Carey V, Huber W, Hahne F. genefilter: methods for filtering genes from high-throughput experiments. Bioconductor 3.7. 2018; R package version 1.62.0. https://doi.org/10.18129/B9.bioc.genefilter.
3. MacDonald JW. affycoretools: Functions useful for those doing repetitive analyses with Affymetrix GeneChips. Bioconductor 3.7. 2008; R package version 1.52.0. https://doi.org/10.18129/B9.bioc.affycoretools.
4. Ritchie ME, Phipson B, Wu D, Hu Y, Law CW, Shi W, Smyth GK. limma powers differential expression analyses for RNA-sequencing and microarray studies. Nucleic Acids Res. 2015; 43:e47. https://doi.org/10.1093/nar/gkv007.
5. Huntley MA, Larson JL, Chaivorapol C, Becker G, Lawrence M, Hackney JA, Kaminker JS. ReportingTools: an automate d result processing and presentation toolkit for high-throughput genomic analyses. Bioinformatics. 2013; 29:3220–21.
6. Leek JT, Johnson WE, Parker HS, Fertig EJ, Jaffe AE, Storey JD. sva: Surrogate Variable Analysis. Bioconductor. 2018: R package version 3.28.0. https://doi.org/10.18129/B9.bioc.sva.
7. Warnes GR, Bolker B, Bonebakker L, Gentleman R, Liaw WH, Lumley T, Maechler M, Magnusson A, Moeller S, Schwartz M, et al. gplots: Various R Programming Tools for Plotting Data. 2016. https://CRAN.R-project.org/package=gplots.
8. Pau G. hwriter: HTML Writer - Outputs R objects in HTML format. 2014. https://cran.r-project.org/web/packages/hwriter/hwriter.pdf.
9. Carvalho B. pd.hugene.2.1.st: Platform Design Info for Affymetrix HuGene-2_1-st. Bioconductor 3.7. 2015: R package version 3.14.1. https://doi.org/10.18129/B9.bioc.pd.hugene.2.1.st.
10. MacDonald JW. hugene21sttranscriptcluster.db: Affymetrix hugene21 annotation data (chip hugene21sttranscriptcluster). Bioconductor 3.7. 2017: R package version 8.7.0. https://doi.org/10.18129/B9.bioc.hugene21sttranscriptcluster.db.
11. Johnson WE, Li C, Rabinovic A. Adjusting batch effects in microarray expression data using empirical Bayes methods. Biostatistics. 2007; 8:118–127.
12. Smyth GK. Linear models and empirical bayes methods for assessing differential expression in microarray experiments. Stat Appl Genet Mol Biol. 2004; 3:1–25.
13. Benjamini Y, Hochberg Y. Controlling the False Discovery Rate: A Practical and Powerful Approach to Multiple Testing. Journal of the Royal Statistical Society. Series B (Methodological). 1995; 57: 289-300.
14. Subramanian A, Tamayo P, Mootha VK, Mukherjee S, Ebert BL, Gillette MA, Paulovich A, Pomeroy SL, Golub TR, Lander ES, Mesirov JP. Gene set enrichment analysis: a knowledge-based approach for interpreting genome-wide expression profiles Proc Natl Acad Sci U S A. 2005; 102:15545–15550.
15. Donato M, Xu Z, Tomoiaga A, Granneman JG, Mackenzie RG, Bao R, Than NG, Westfall PH, Romero R, Draghici S. Analysis and correction of crosstalk effects in pathway analysis. Genome research. 2013; 23:1885–1893.
16. Draghici S, Khatri P, Tarca AL, Amin K, Done A, Voichita C, Georgescu C, Romero R. A systems biology approach for pathway level analysis. Genome research. 2007; 17:1537–1545.

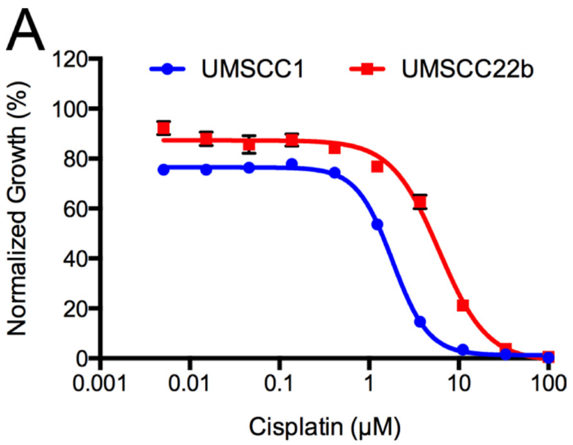

Supplementary Figure 1: IC_{50} analysis of cisplatin-treated UM-SCC-1 and UM-SCC-22b cells. (**A**) UM-SCC-1 (blue circles) and UM-SCC-22b (red squares) were plated in 96-well plates and treated for 3 days with increasing concentration of cisplatin. Growth was measured by Alamar Blue assay, normalized to untreated cells (100%) and wells containing only media (0%), and curve-fitted with non-linear regression.

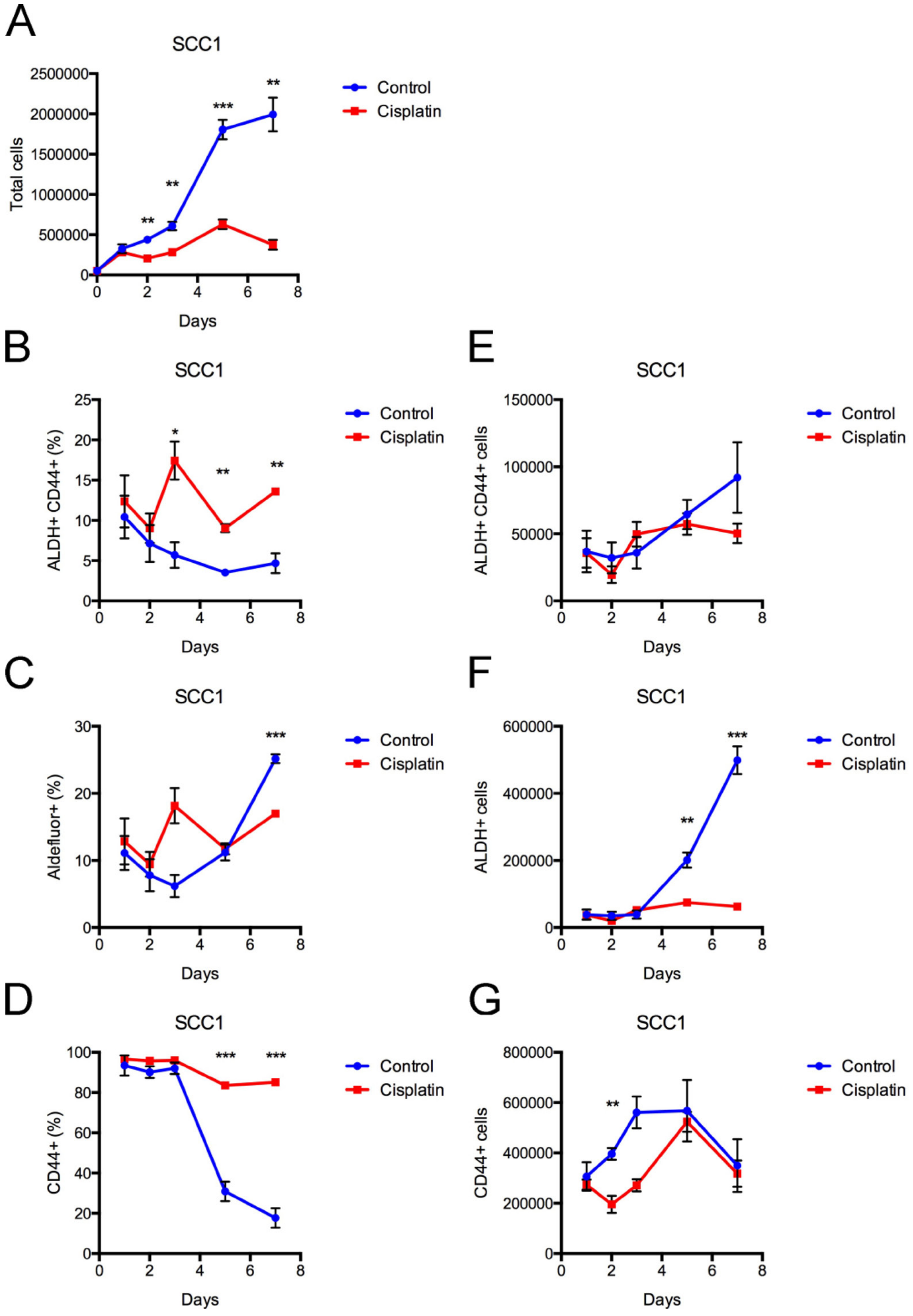

Supplementary Figure 2: Response of UM-SCC-1 cells to cisplatin treatment. (**A**) Total cell counts for control (blue circles) and 2μM cisplatin (red squares). The frequency of (**B**) $ALDH^{high}CD44^{high}$, (**C**) $ALDH^{high}$, and (**D**) $CD44^{high}$ cells using gating based on DEAB sample. The absolute number of (**E**) $ALDH^{high}CD44^{high}$, (**F**) $ALDH^{high}$ and (**G**) $CD44^{high}$ cells. Mean ± standard deviation. $N = 3$. Two-sided t-tests with Bonferroni multiple testing correction. $^{*}p < 0.05$, $^{**}p < 0.01$, $^{***}p < 0.001$, $^{****}p < 0.0001$.

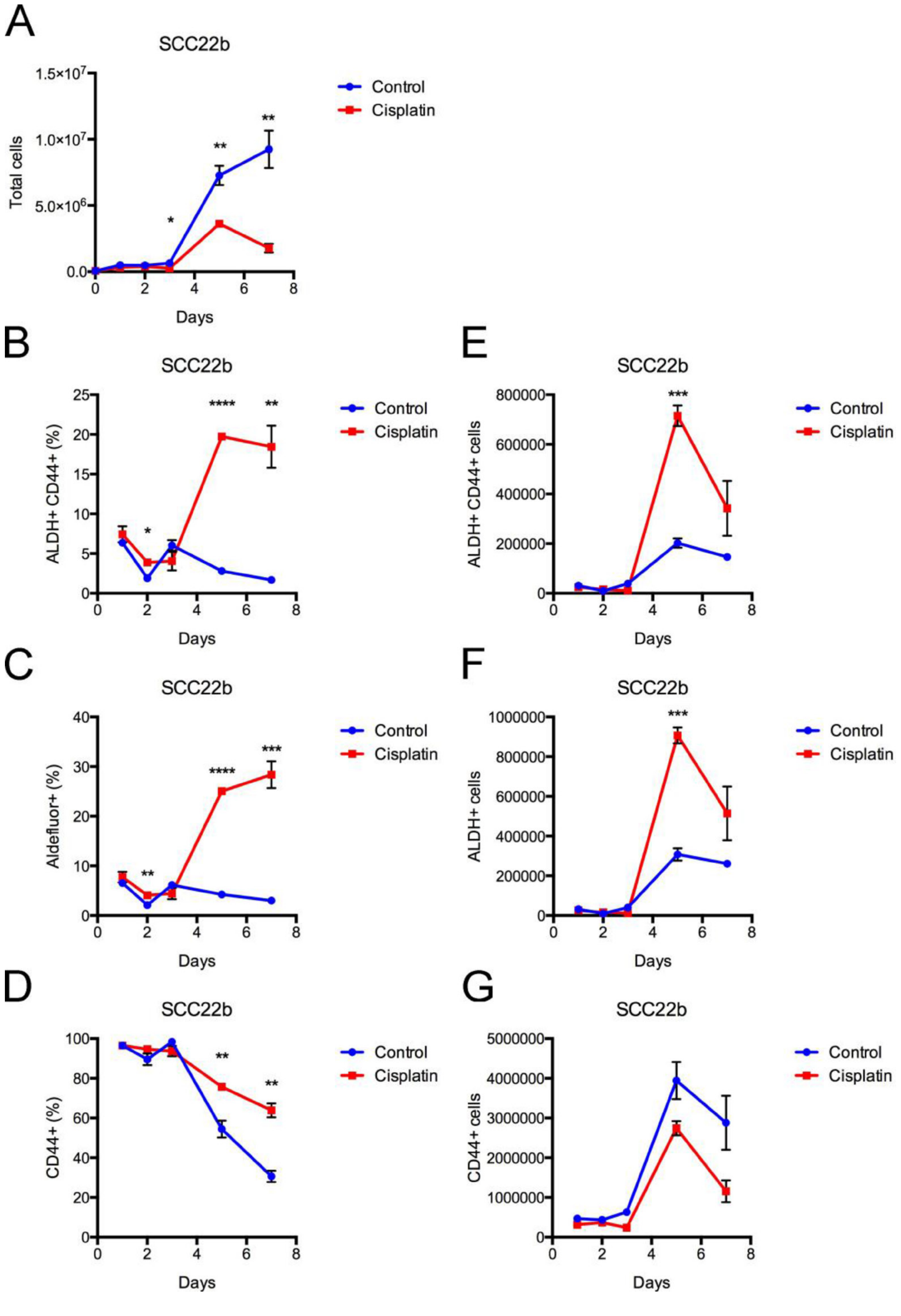

Supplementary Figure 3: Response of UM-SCC-22b cells to cisplatin treatment. (**A**) Total cell counts for control (blue circles) and 2μM cisplatin (red squares). The frequency of (**B**) $ALDH^{high}CD44^{high}$, (**C**) $ALDH^{high}$, and (**D**) $CD44^{high}$ cells using gating based on DEAB sample. The absolute number of (**E**) $ALDH^{high}CD44^{high}$, (**F**) $ALDH^{high}$, and (**G**) $CD44^{high}$ cells. Mean ± standard deviation. $N = 3$. Two-sided t-tests with Bonferroni multiple testing correction. $^{*}p < 0.05$, $^{**}p < 0.01$, $^{***}p < 0.001$, $^{****}p < 0.0001$.

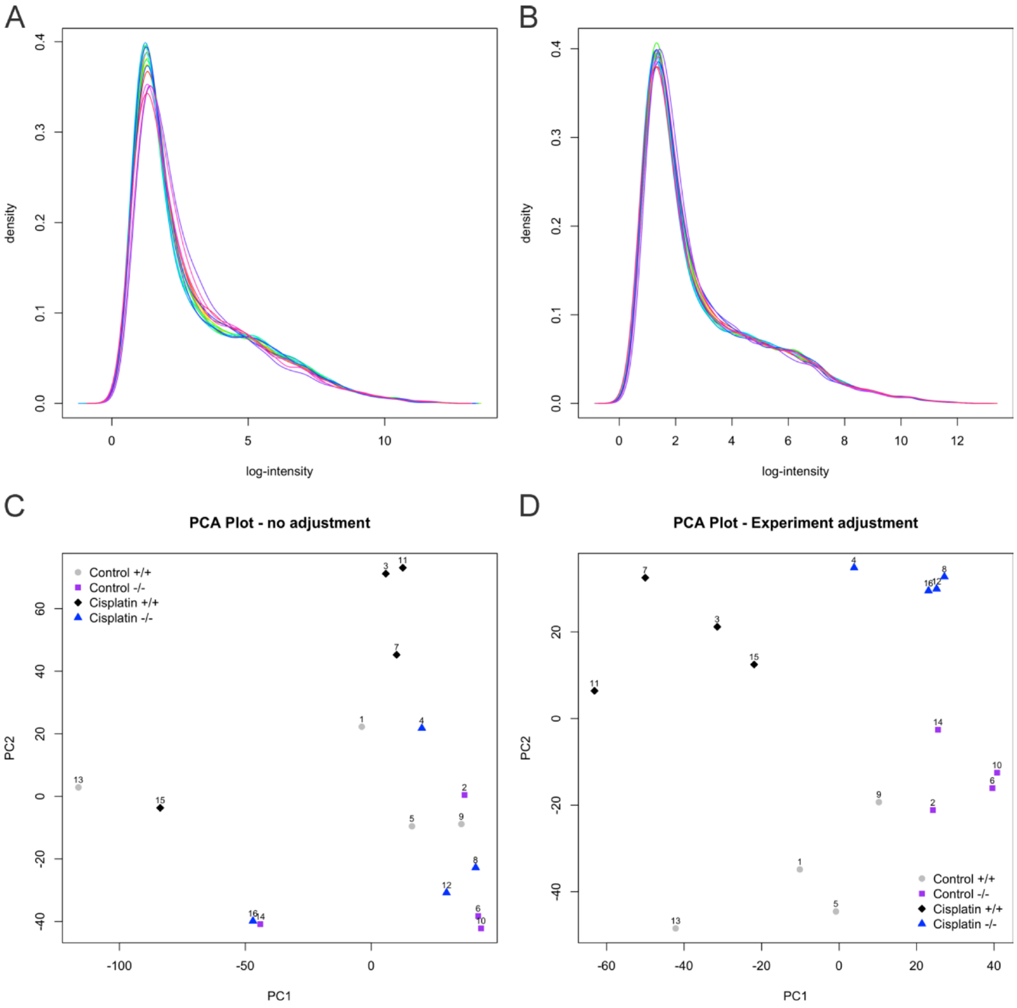

Supplementary Figure 4: Adjustment of microarray batch variable. (**A**, **B**) Histogram analysis of 16 microarray samples (A) before or (B) after 'Combat' batch adjustment. (**C**, **D**) Principal component analysis of 16 microarray samples (C) before or (D) after 'Combat' batch adjustment. Samples 13-16 are the pilot microarray and represent the largest variance.

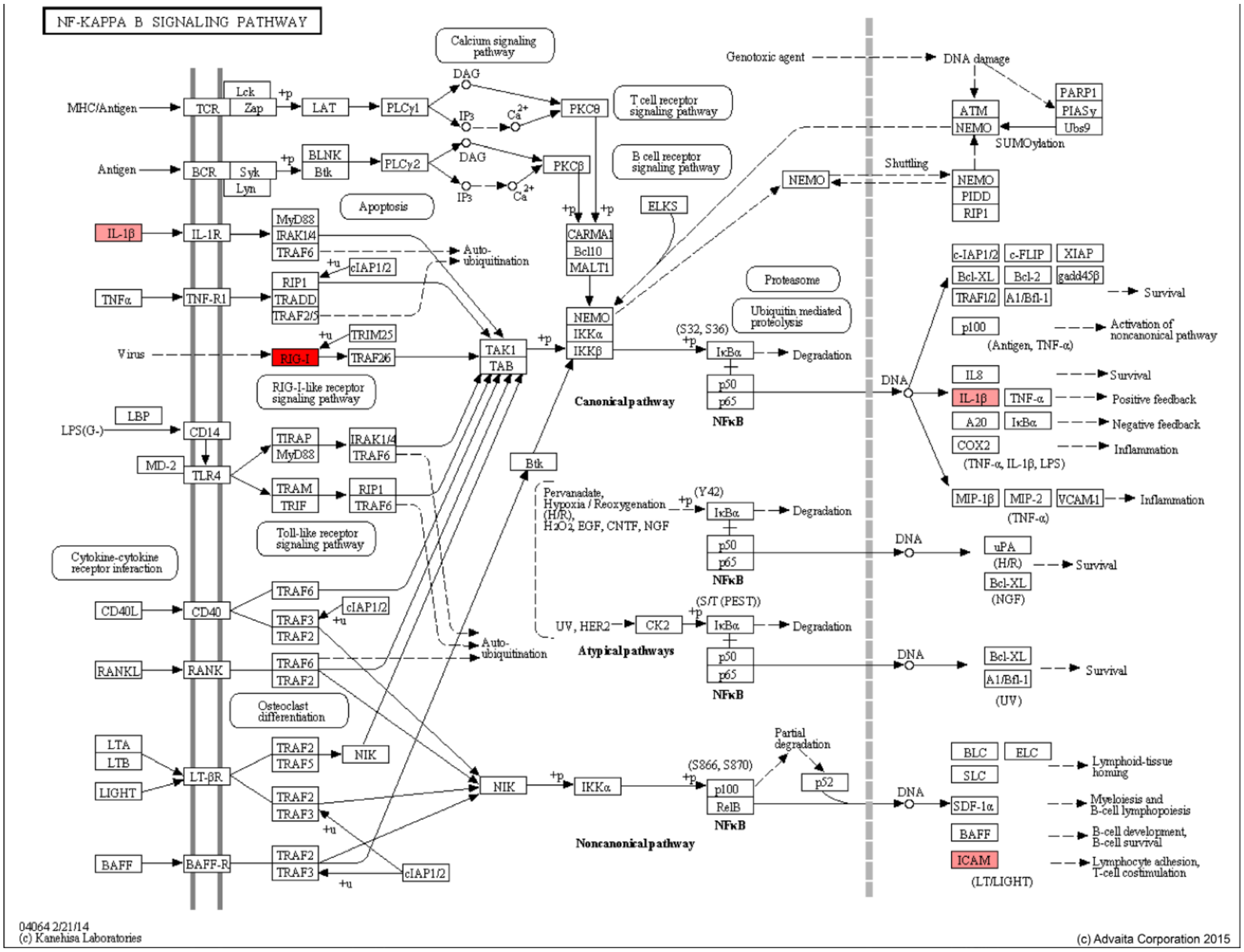

Supplementary Figure 5: NF-κB Signaling Pathway Enriched via iPathway Analysis. iPathway analysis identified NF-κB signaling (via KEGG database) enriched in the comparison of cisplatin ALDH^{high}CD44^{high} vs. control ALDH^{high}CD44^{high}. Genes enriched in cisplatin ALDH^{high}CD44^{high} are depicted in red; genes enriched in control ALDH^{high}CD44^{high} are depicted in blue.

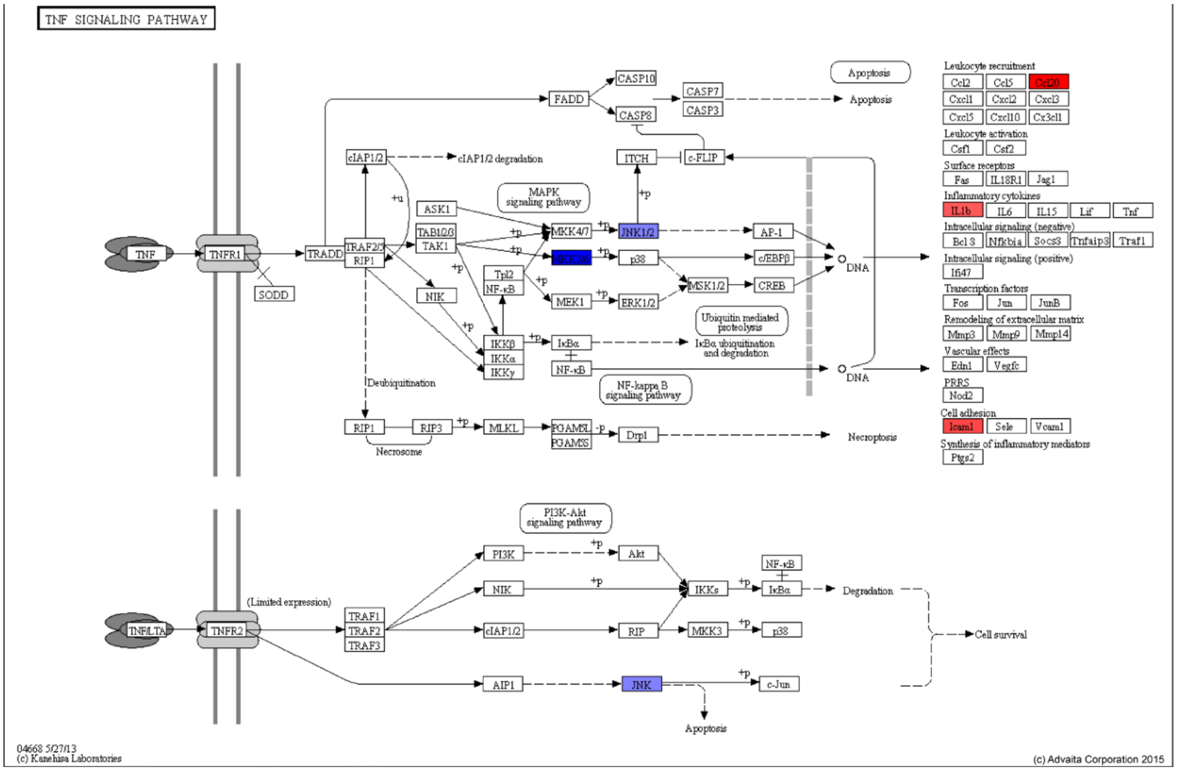

Supplementary Figure 6: TNF Signaling Pathway Enriched via iPathway Analysis. iPathway analysis identified TNF signaling (via KEGG database) enriched in the comparison of cisplatin ALDH^{high}CD44^{high} vs. control ALDH^{high}CD44^{high}. Genes enriched in cisplatin ALDH^{high}CD44^{high} are depicted in red; genes enriched in control ALDH^{high}CD44^{high} are depicted in blue.

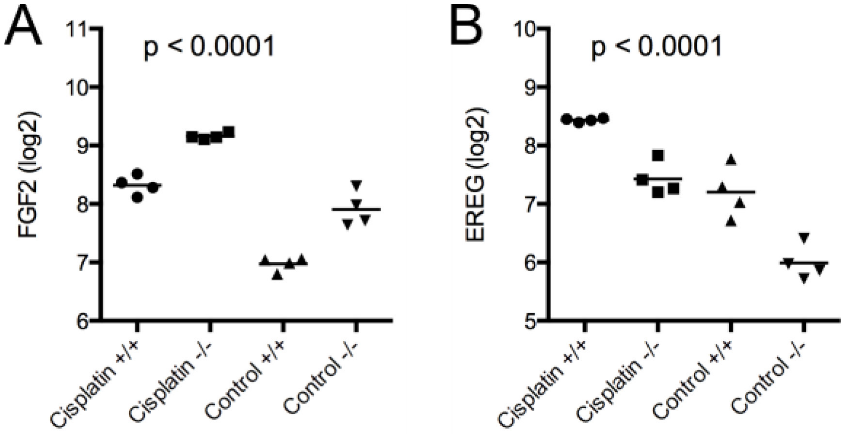

Supplementary Figure 7: Evaluation of FGF2 and EREG from microarray. Log2 expression values from the microarrays for (**A**) FGF2 and (**B**) EREG. *P*-values represent overall one-way ANOVA.

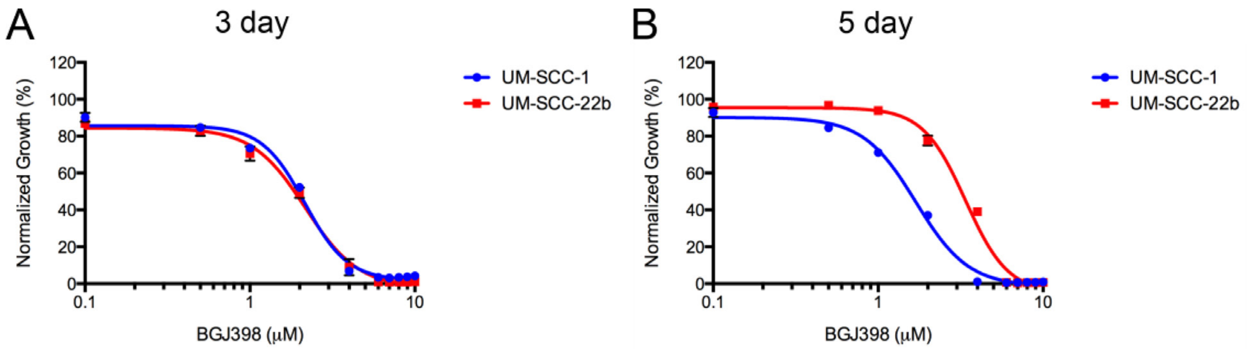

Supplementary Figure 8: BGJ398 IC_{50} analysis. 1000 UM-SCC-1 (blue circles) or 2000 UM-SCC-22b (red squares) cells were plated for (**A**) 3 or (**B**) 5 days in 96-well plates. BGJ398 was added for a final concentration from 0.1 to 10μM. Growth was measured by Alamar Blue assay, normalized to untreated cells (100%) and wells containing only media (0%), and curve-fitted with non-linear regression.

Supplementary Table 1: RNA integrity of microarray samples

Exp	Treatment	Population	RIN	RNA (ng)	$^{\#}$cells	RNA (pg)/cell
Pilot	Control	$ALDH^{high}CD44^{high}$	9.00	88	8,960	9.8
Pilot	Control	$ALDH^{low}CD44^{low}$	9.90	2,561	426,880	6.0
Pilot	Cisplatin	$ALDH^{high}CD44^{high}$	9.20	159	10,700	14.9
Pilot	Cisplatin	$ALDH^{low}CD44^{low}$	9.80	4,583	376,980	12.2
1	Control	$ALDH^{high}CD44^{high}$	9.50	79	2,770	28.4
1	Control	$ALDH^{low}CD44^{low}$	8.10	551	89,250	6.2
1	Cisplatin	$ALDH^{high}CD44^{high}$	8.60	70	2,730	25.7
1	Cisplatin	$ALDH^{low}CD44^{low}$	8.20	85	8,650	9.8
2	Control	$ALDH^{high}CD44^{high}$	9.00	163	13,250	12.3
2	Control	$ALDH^{low}CD44^{low}$	10.00	6,488	766,900	8.5
2	Cisplatin	$ALDH^{high}CD44^{high}$	9.00	104	2,170	48.0
2	Cisplatin	$ALDH^{low}CD44^{low}$	10.00	2,183	325,060	6.7
3	Control	$ALDH^{high}CD44^{high}$	9.20	300	32,800	9.1
3	Control	$ALDH^{low}CD44^{low}$	10.00	9,126	1,090,000	8.4
3	Cisplatin	$ALDH^{high}CD44^{high}$	7.80	117	3,700	31.7
3	Cisplatin	$ALDH^{low}CD44^{low}$	10.00	7,501	667,840	11.2
4	Control	$ALDH^{high}CD44^{high}$	8.50	26	405	63.3
4	Control	$ALDH^{low}CD44^{low}$	8.40	614	115,130	5.3
4	Cisplatin	$ALDH^{high}CD44^{high}$	9.10	45	2,650	17.2
4	Cisplatin	$ALDH^{low}CD44^{low}$	10.00	4,423	512,470	8.6

The RNA Integrity Number (RIN), total RNA (ng), number of FACS-collected cells, and amount of RNA (pg) per cell for the FACS experiments used for microarray analysis.

Supplementary Table 2: Leading Edge Analysis of Gene Sets Enriched in Cisplatin ALDH^{high}CD44^{high} vs. Control ALDH^{high}CD44^{high}

Gene	HALLMARK INTERFERON ALPHA RESPONSE	HALLMARK TNFα SIGNALING VIA NFKB	HALLMARK IL6 JAK STAT3 SIGNALING
CXCL10	X	X	X
CXCL11	X	X	X
IRF1	X	X	X
RIPK2	X	X	
IL15RA		X	X
IL6		X	X
IL4R	X		X
LY6E	X	X	
BST2	X	X	
RTP4		X	X
IL15	X		X
IRF7	X		X
IFIT2		X	X
IFIH1		X	X
IL1B		X	X
ICAM1		X	X

Gene Set Enrichment Analysis was performed for cisplatin ALDH^{high}CD44^{high} vs. control ALDH^{high}CD44^{high}. Leading Edge Analysis was performed using three of the "Hallmark" gene sets enriched in cisplatin ALDH^{high}CD44^{high} cells. Some of the genes are in common within the three Hallmark gene sets, Interferon Alpha Response, TNFα Signaling Via NF-κB, and IL6-JAK-STAT3 Signaling.

Supplementary Table 3: iPathway analysis of Cisplatin ALDH^{high}CD44^{high} vs. Control ALDH^{high}CD44^{high}

Pathway Name	*P*-value	Bonferroni Adj *P*-value	FDR Adj *P*-value
Influenza A	2.49E-07	3.61E-05	2.33E-05
Herpes simplex infection	3.21E-07	4.65E-05	2.33E-05
Measles	9.42E-05	0.013654	0.004551
Malaria	0.000209	0.030366	0.007591
Hepatitis C	0.000382	0.055426	0.011085
TNF signaling pathway	0.005782	0.838410	0.126821
NF-kappa B signaling pathway	0.007406	1	0.126821
Epstein-Barr virus infection	0.007813	1	0.126821
Cytosolic DNA-sensing pathway	0.007872	1	0.126821
Osteoclast differentiation	0.012901	1	0.185015
ErbB signaling pathway	0.014036	1	0.185015
Toll-like receptor signaling pathway	0.020263	1	0.236966
Hepatitis B	0.021245	1	0.236966
RIG-I-like receptor signaling pathway	0.023531	1	0.243713
Nicotinate and nicotinamide metabolism	0.026829	1	0.245565
Leishmaniasis	0.027410	1	0.245565
Pentose phosphate pathway	0.028790	1	0.245565
Amoebiasis	0.036477	1	0.293841
African trypanosomiasis	0.044945	1	0.343000

Pathway analysis was performed with iPathway using the genes with adjusted *P*-value < 0.05 in the cisplatin ALDH^{high}CD44^{high} vs. control ALDH^{high}CD44^{high} comparison. Unadjusted p-value, bonferonni and FDR adjusted *p*-values are shown for the pathways with an unadjusted *p*-value < 0.05.

Supplementary Table 4: Genes higher in cisplatin ALDH^{high}CD44^{high} cells vs. control ALDH^{high}CD44^{high} cells. RMA normalized microarray data was fitted to a linear model and initial statistics were determined using an empirical Bayesian model. Multiple testing comparisons were adjusted using Benjamini and Hochberg (aka FDR). Probes with an adjusted p-value < 0.05 were considered statistically significant. The Affymetrix ProbeID, EntrezID, HUGO gene symbol, gene name, log2 fold change, and adjusted p-value are shown for the 115 genes higher in cisplatin ALDH^{high}CD44^{high} vs. control ALDH^{high}CD44^{high}. See Supplementary_Table_4

Supplementary Table 5: Genes lower in cisplatin ALDH^{high}CD44^{high} cells vs. control ALDH^{high}CD44^{high} cells. RMA normalized microarray data was fitted to a linear model and initial statistics were determined using an empirical Bayesian model. Multiple testing comparisons were adjusted using Benjamini and Hochberg (aka FDR). Probes with an adjusted p-value < 0.05 were considered statistically significant. The Affymetrix ProbeID, EntrezID, HUGO gene symbol, gene name, log2 fold change, and adjusted p-value are shown for the 116 genes lower in cisplatin ALDH^{high}CD44^{high} vs. control ALDH^{high}CD44^{high}. See Supplementary_Table_5

Supplementary Table 6: IC$_{50}$ analysis of BGJ398-treated UM-SCC-1 and UM-SCC-22b cells

	3 day		5 day	
**IC50 (µM)**	**UM-SCC-1**	**UM-SCC-22b**	**UM-SCC-1**	**UM-SCC-22b**
Average	2.211	2.210	1.909	3.569
Std. Deviation	0.084	0.056	0.308	0.258
Std. Error of Mean	0.060	0.040	0.218	0.183

The average BGJ398 IC$_{50}$ (µM), standard deviation, and standard error of mean from representative experiment of 2 plates/experiment with 3 wells per dose. Cells were treated for 3 or 5 days with 0.1 to 10µM BGJ398 and growth measured by Alamar Blue assay.
