# Supplementary material for: FGFR signaling regulates resistance of head and neck cancer stem cells to cisplatin
Source: Oncotarget. 2018 May 18;9(38):25148–65. doi: 10.18632/oncotarget.25358 (PMC5982758; doi:10.18632/oncotarget.25358)
Supplement: Supplementary file 2 [file oncotarget-09-25148-s002.docx]

**Supplementary Table 4: Genes higher in cisplatin ALDH^high^CD44^high^ cells vs. control ALDH^high^CD44^high^ cells**

| PROBE ID | ENTREZ ID | SYMBOL | GENENAME | Log FC | adj. P.Val |
| --- | --- | --- | --- | --- | --- |
| 17104821 | 100130361 | CXorf49 | chromosome X open reading frame 49 | 2.080 | 0.0004 |
| 16876764 | 91543 | RSAD2 | radical S-adenosyl methionine domain containing 2 | 2.069 | 0.0044 |
| 16736453 | 6289 | SAA2 | serum amyloid A2 | 1.984 | 0.0298 |
| 16757373 | 4939 | OAS2 | 2'-5'-oligoadenylate synthetase 2, 69/71kDa | 1.963 | 0.0008 |
| 16666485 | 10964 | IFI44L | interferon-induced protein 44-like | 1.881 | 0.0002 |
| 17104825 | 100130361 | CXorf49 | chromosome X open reading frame 49 | 1.872 | 0.0004 |
| 17111971 | 100130361 | CXorf49 | chromosome X open reading frame 49 | 1.872 | 0.0004 |
| 16707180 | 3433 | IFIT2 | interferon-induced protein with tetratricopeptide repeats 2 | 1.828 | 0.0005 |
| 16787814 | 3429 | IFI27 | interferon, alpha-inducible protein 27 | 1.758 | 0.0009 |
| 16666509 | 10561 | IFI44 | interferon-induced protein 44 | 1.729 | 0.0002 |
| 16873060 | 5329 | PLAUR | plasminogen activator, urokinase receptor | 1.706 | 0.0002 |
| 17093090 | 23586 | DDX58 | DEAD (Asp-Glu-Ala-Asp) box polypeptide 58 | 1.669 | 0.0002 |
| 16722562 | 6288 | SAA1 | serum amyloid A1 | 1.615 | 0.0034 |
| 16830202 | 54739 | XAF1 | XIAP associated factor 1 | 1.603 | 0.0041 |
| 17055786 | 541472 | LOC541472 | uncharacterized LOC541472 | 1.592 | 0.0373 |
| 16852871 | 5055 | SERPINB2 | serpin peptidase inhibitor, clade B (ovalbumin), member 2 | 1.536 | 0.0037 |
| 16705329 | 84665 | MYPN | myopalladin | 1.512 | 0.0116 |
| 16859205 | 652995 | UCA1 | urothelial cancer associated 1 (non-protein coding) | 1.503 | 0.0008 |
| 16657594 | 9636 | ISG15 | ISG15 ubiquitin-like modifier | 1.476 | 0.0013 |
| 16707184 | 3437 | IFIT3 | interferon-induced protein with tetratricopeptide repeats 3 | 1.457 | 0.0002 |
| 16707196 | 3434 | IFIT1 | interferon-induced protein with tetratricopeptide repeats 1 | 1.394 | 0.0004 |
| 16922959 | 4600 | MX2 | MX dynamin-like GTPase 2 | 1.386 | 0.0380 |
| 16968735 | 55008 | HERC6 | HECT and RLD domain containing E3 ubiquitin protein ligase family member 6 | 1.355 | 0.0006 |
| 16967831 | 255324 | EPGN | epithelial mitogen | 1.344 | 0.0011 |
| 16970404 | 2247 | FGF2 | fibroblast growth factor 2 (basic) | 1.320 | 0.0006 |
| 16771417 | 8638 | OASL | 2'-5'-oligoadenylate synthetase-like | 1.306 | 0.0245 |
| 16684080 | 2537 | IFI6 | interferon, alpha-inducible protein 6 | 1.300 | 0.0036 |
| 16967843 | 2069 | EREG | epiregulin | 1.289 | 0.0116 |
| 16672349 | 391109 | OR10K1 | olfactory receptor, family 10, subfamily K, member 1 | 1.223 | 0.0223 |
| 16976868 | 685 | BTC | betacellulin | 1.199 | 0.0451 |
| 16931766 | 113730 | KLHDC7B | kelch domain containing 7B | 1.197 | 0.0020 |
| 16764791 | 144501 | KRT80 | keratin 80 | 1.189 | 0.0022 |
| 17010760 | 4907 | NT5E | 5'-nucleotidase, ecto (CD73) | 1.183 | 0.0114 |
| 17082081 | 78998 | RHPN1-AS1 | RHPN1 antisense RNA 1 (head to head) | 1.161 | 0.0427 |
| 16968765 | 51191 | HERC5 | HECT and RLD domain containing E3 ubiquitin protein ligase 5 | 1.144 | 0.0111 |
| 16984029 | 100506406 | LOC100506406 | uncharacterized LOC100506406 | 1.096 | 0.0432 |
| 16743926 | 59082 | CARD18 | caspase recruitment domain family, member 18 | 1.086 | 0.0275 |
| 17117441 | 723788 | MIG7 | mig-7 | 1.085 | 0.0084 |
| 16757324 | 4938 | OAS1 | 2'-5'-oligoadenylate synthetase 1, 40/46kDa | 1.080 | 0.0005 |
| 16837418 | 6662 | SOX9 | SRY (sex determining region Y)-box 9 | 1.051 | 0.0223 |
| 16923031 | 4599 | MX1 | MX dynamin-like GTPase 1 | 1.050 | 0.0008 |
| 16990767 | 404203 | SPINK6 | serine peptidase inhibitor, Kazal type 6 | 1.039 | 0.0104 |
| 16778559 | 94240 | EPSTI1 | epithelial stromal interaction 1 (breast) | 1.032 | 0.0011 |
| 16697095 | 4688 | NCF2 | neutrophil cytosolic factor 2 | 1.025 | 0.0150 |
| 16995601 | 2533 | FYB | FYN binding protein | 1.021 | 0.0209 |
| 16976894 | 100506253 | LOC100506253 | uncharacterized LOC100506253 | 1.001 | 0.0180 |
| 16891774 | 6364 | CCL20 | chemokine (C-C motif) ligand 20 | 1.000 | 0.0305 |
| 16679480 | 100616343 | MIR4677 | microRNA 4677 | 0.987 | 0.0474 |
| 16752185 | 196410 | METTL7B | methyltransferase like 7B | 0.979 | 0.0451 |
| 16740630 | 8061 | FOSL1 | FOS-like antigen 1 | 0.978 | 0.0305 |
| 16981219 | 55601 | DDX60 | DEAD (Asp-Glu-Ala-Asp) box polypeptide 60 | 0.953 | 0.0107 |
| 16981266 | 91351 | DDX60L | DEAD (Asp-Glu-Ala-Asp) box polypeptide 60-like | 0.941 | 0.0008 |
| 16698304 | 127845 | GOLT1A | golgi transport 1A | 0.940 | 0.0169 |
| 16907412 | 7341 | SUMO1 | small ubiquitin-like modifier 1 | 0.936 | 0.0122 |
| 16852858 | 8710 | SERPINB7 | serpin peptidase inhibitor, clade B (ovalbumin), member 7 | 0.928 | 0.0446 |
| 16870200 | 684 | BST2 | bone marrow stromal cell antigen 2 | 0.926 | 0.0181 |
| 16734491 | 7262 | PHLDA2 | pleckstrin homology-like domain, family A, member 2 | 0.920 | 0.0143 |
| 16916682 | 83959 | SLC4A11 | solute carrier family 4, sodium borate transporter, member 11 | 0.898 | 0.0111 |
| 16757347 | 4940 | OAS3 | 2'-5'-oligoadenylate synthetase 3, 100kDa | 0.893 | 0.0223 |
| 17004836 | 221710 | SMIM13 | small integral membrane protein 13 | 0.893 | 0.0440 |
| 16984730 | 10468 | FST | follistatin | 0.878 | 0.0169 |
| 16851397 | 5932 | RBBP8 | retinoblastoma binding protein 8 | 0.873 | 0.0124 |
| 16870990 | 100505851 | LOC100505851 | uncharacterized LOC100505851 | 0.857 | 0.0083 |
| 16804490 | 3669 | ISG20 | interferon stimulated exonuclease gene 20kDa | 0.850 | 0.0176 |
| 17089525 | 3934 | LCN2 | lipocalin 2 | 0.849 | 0.0423 |
| 16750792 | 79962 | DNAJC22 | DnaJ (Hsp40) homolog, subfamily C, member 22 | 0.849 | 0.0264 |
| 16884280 | 440894 | LINC01123 | long intergenic non-protein coding RNA 1123 | 0.841 | 0.0239 |
| 16859281 | 84941 | HSH2D | hematopoietic SH2 domain containing | 0.828 | 0.0067 |
| 17062355 | 168433 | RNF133 | ring finger protein 133 | 0.817 | 0.0274 |
| 16844044 | 619505 | SNORA21 | small nucleolar RNA, H/ACA box 21 | 0.815 | 0.0446 |
| 16781873 | 28673 | TRAV12-2 | T cell receptor alpha variable 12-2 | 0.796 | 0.0244 |
| 16884523 | 6574 | SLC20A1 | solute carrier family 20 (phosphate transporter), member 1 | 0.791 | 0.0104 |
| 16774405 | 29103 | DNAJC15 | DnaJ (Hsp40) homolog, subfamily C, member 15 | 0.780 | 0.0202 |
| 16958124 | 83666 | PARP9 | poly (ADP-ribose) polymerase family, member 9 | 0.773 | 0.0223 |
| 16964735 | 6286 | S100P | S100 calcium binding protein P | 0.771 | 0.0264 |
| 16977502 | 51316 | PLAC8 | placenta-specific 8 | 0.766 | 0.0257 |
| 16937503 | 442075 | EMC3-AS1 | EMC3 antisense RNA 1 | 0.753 | 0.0131 |
| 16909021 | 5270 | SERPINE2 | serpin peptidase inhibitor, clade E (nexin, plasminogen activator inhibitor type 1), member 2 | 0.744 | 0.0161 |
| 16764114 | 7480 | WNT10B | wingless-type MMTV integration site family, member 10B | 0.730 | 0.0265 |
| 16798398 | 100036564 | SNORD115-27 | small nucleolar RNA, C/D box 115-27 | 0.729 | 0.0380 |
| 17023697 | 9288 | TAAR3 | trace amine associated receptor 3 (gene/pseudogene) | 0.725 | 0.0312 |
| 16858137 | 3383 | ICAM1 | intercellular adhesion molecule 1 | 0.722 | 0.0410 |
| 16849556 | 7077 | TIMP2 | TIMP metallopeptidase inhibitor 2 | 0.722 | 0.0008 |
| 17059771 | 54809 | SAMD9 | sterile alpha motif domain containing 9 | 0.716 | 0.0082 |
| 16978417 | 64116 | SLC39A8 | solute carrier family 39 (zinc transporter), member 8 | 0.709 | 0.0371 |
| 16826212 | 388272 | C16orf87 | chromosome 16 open reading frame 87 | 0.707 | 0.0116 |
| 16796694 | 7453 | WARS | tryptophanyl-tRNA synthetase | 0.707 | 0.0342 |
| 16904365 | 64135 | IFIH1 | interferon induced with helicase C domain 1 | 0.704 | 0.0165 |
| 16707202 | 24138 | IFIT5 | interferon-induced protein with tetratricopeptide repeats 5 | 0.692 | 0.0275 |
| 16781536 | 4860 | PNP | purine nucleoside phosphorylase | 0.681 | 0.0447 |
| 16817721 | 253982 | ASPHD1 | aspartate beta-hydroxylase domain containing 1 | 0.675 | 0.0394 |
| 16727650 | 29984 | RHOD | ras homolog family member D | 0.661 | 0.0121 |
| 16774669 | 55270 | NUDT15 | nudix (nucleoside diphosphate linked moiety X)-type motif 15 | 0.660 | 0.0150 |
| 16901986 | 3553 | IL1B | interleukin 1, beta | 0.654 | 0.0424 |
| 16826230 | 81831 | NETO2 | neuropilin (NRP) and tolloid (TLL)-like 2 | 0.647 | 0.0432 |
| 16720085 | 8519 | IFITM1 | interferon induced transmembrane protein 1 | 0.639 | 0.0274 |
| 16799492 | 100505573 | INAFM2 | InaF-motif containing 2 | 0.634 | 0.0430 |
| 16677153 | 84791 | LINC00467 | long intergenic non-protein coding RNA 467 | 0.633 | 0.0438 |
| 16955225 | 26059 | ERC2 | ELKS/RAB6-interacting/CAST family member 2 | 0.632 | 0.0424 |
| 16866263 | 100507739 | ERVK3-1 | endogenous retrovirus group K3, member 1 | 0.626 | 0.0420 |
| 16906534 | 6772 | STAT1 | signal transducer and activator of transcription 1, 91kDa | 0.616 | 0.0243 |
| 16973879 | 55646 | LYAR | Ly1 antibody reactive | 0.609 | 0.0116 |
| 16753641 | 8091 | HMGA2 | high mobility group AT-hook 2 | 0.592 | 0.0442 |
| 16833689 | 284106 | CISD3 | CDGSH iron sulfur domain 3 | 0.589 | 0.0489 |
| 16742963 | 54843 | SYTL2 | synaptotagmin-like 2 | 0.573 | 0.0275 |
| 17088185 | 9128 | PRPF4 | pre-mRNA processing factor 4 | 0.567 | 0.0493 |
| 16917030 | 51605 | TRMT6 | tRNA methyltransferase 6 homolog (S. cerevisiae) | 0.565 | 0.0223 |
| 16919022 | 25939 | SAMHD1 | SAM domain and HD domain 1 | 0.513 | 0.0216 |
| 17093147 | 54840 | APTX | aprataxin | 0.511 | 0.0478 |
| 16705474 | 9188 | DDX21 | DEAD (Asp-Glu-Ala-Asp) box helicase 21 | 0.504 | 0.0430 |
| 16825794 | 79077 | DCTPP1 | dCTP pyrophosphatase 1 | 0.496 | 0.0338 |
| 16749398 | 23012 | STK38L | serine/threonine kinase 38 like | 0.486 | 0.0404 |
| 16777896 | 10808 | HSPH1 | heat shock 105kDa/110kDa protein 1 | 0.471 | 0.0389 |
| 16784157 | 54331 | GNG2 | guanine nucleotide binding protein (G protein), gamma 2 | 0.458 | 0.0451 |
| 16896442 | 5610 | EIF2AK2 | eukaryotic translation initiation factor 2-alpha kinase 2 | 0.423 | 0.0404 |

RMA normalized microarray data was fitted to a linear model and initial statistics were determined using an empirical Bayesian model. Multiple testing comparisons were adjusted using Benjamini and Hochberg (aka FDR). Probes with an adjusted p-value <0.05 were considered statistically significant. The Affymetrix ProbeID, EntrezID, HUGO gene symbol, gene name, log2 fold change, and adjusted p-value are shown for the 115 genes higher in cisplatin ALDH^high^CD44^high^ vs. control ALDH^high^CD44^high^.
