# Supplementary material for: FGFR signaling regulates resistance of head and neck cancer stem cells to cisplatin
Source: Oncotarget. 2018 May 18;9(38):25148–65. doi: 10.18632/oncotarget.25358 (PMC5982758; doi:10.18632/oncotarget.25358)
Supplement: Supplementary file 3 [file oncotarget-09-25148-s003.docx]

**Supplementary Table 5: Genes lower in cisplatin ALDH^high^CD44^high^ cells vs. control ALDH^high^CD44^high^ cells**

| PROBE ID | ENTREZ ID | SYMBOL | GENENAME | Log FC | adj. P.Val |
| --- | --- | --- | --- | --- | --- |
| 16765029 | 3848 | KRT1 | keratin 1 | -2.514 | 0.0044 |
| 16859090 | 23581 | CASP14 | caspase 14, apoptosis-related cysteine peptidase | -1.983 | 0.0176 |
| 16693249 | 284486 | THEM5 | thioesterase superfamily member 5 | -1.980 | 0.0008 |
| 16698782 | 407026 | MIR29C | microRNA 29c | -1.877 | 0.0104 |
| 17108003 | 633 | BGN | biglycan | -1.844 | 0.0004 |
| 16765080 | 3851 | KRT4 | keratin 4 | -1.484 | 0.0123 |
| 17090713 | 1056 | CEL | carboxyl ester lipase | -1.460 | 0.0430 |
| 16962894 | 100131551 | LINC00887 | long intergenic non-protein coding RNA 887 | -1.399 | 0.0380 |
| 16837348 | 5608 | MAP2K6 | mitogen-activated protein kinase kinase 6 | -1.388 | 0.0056 |
| 16948461 | 6657 | SOX2 | SRY (sex determining region Y)-box 2 | -1.270 | 0.0005 |
| 16808322 | 9677 | PPIP5K1 | diphosphoinositol pentakisphosphate kinase 1 | -1.268 | 0.0029 |
| 16777190 | 10804 | GJB6 | gap junction protein, beta 6, 30kDa | -1.248 | 0.0170 |
| 16819099 | 84290 | CAPNS2 | calpain, small subunit 2 | -1.238 | 0.0338 |
| 16836511 | 284161 | GDPD1 | glycerophosphodiester phosphodiesterase domain containing 1 | -1.154 | 0.0008 |
| 16861882 | 653499 | LGALS7B | lectin, galactoside-binding, soluble, 7B | -1.153 | 0.0451 |
| 16669105 | 126868 | MAB21L3 | mab-21-like 3 (C. elegans) | -1.146 | 0.0020 |
| 16844752 | 3866 | KRT15 | keratin 15 | -1.126 | 0.0008 |
| 16666755 | 9635 | CLCA2 | chloride channel accessory 2 | -1.120 | 0.0259 |
| 17089003 | 169611 | OLFML2A | olfactomedin-like 2A | -1.110 | 0.0009 |
| 17047795 | 948 | CD36 | CD36 molecule (thrombospondin receptor) | -1.109 | 0.0298 |
| 17081401 | 10397 | NDRG1 | N-myc downstream regulated 1 | -1.043 | 0.0298 |
| 16842326 | 218 | ALDH3A1 | aldehyde dehydrogenase 3 family, member A1 | -1.032 | 0.0012 |
| 17020103 | 2941 | GSTA4 | glutathione S-transferase alpha 4 | -1.030 | 0.0060 |
| 16695690 | 257177 | C1orf192 | chromosome 1 open reading frame 192 | -1.023 | 0.0179 |
| 16733985 | 100506211 | MIR210HG | MIR210 host gene (non-protein coding) | -1.022 | 0.0082 |
| 16842297 | 146802 | SLC47A2 | solute carrier family 47 (multidrug and toxin extrusion), member 2 | -0.993 | 0.0308 |
| 16761212 | 9976 | CLEC2B | C-type lectin domain family 2, member B | -0.991 | 0.0104 |
| 16919242 | 9935 | MAFB | v-maf avian musculoaponeurotic fibrosarcoma oncogene homolog B | -0.990 | 0.0223 |
| 17047330 | 81554 | WBSCR16 | Williams-Beuren syndrome chromosome region 16 | -0.972 | 0.0104 |
| 16731773 | 56649 | TMPRSS4 | transmembrane protease, serine 4 | -0.960 | 0.0122 |
| 16996946 | 653188 | GUSBP3 | glucuronidase, beta pseudogene 3 | -0.942 | 0.0114 |
| 16779199 | 2974 | GUCY1B2 | guanylate cyclase 1, soluble, beta 2 (pseudogene) | -0.924 | 0.0451 |
| 17025844 | 7058 | THBS2 | thrombospondin 2 | -0.919 | 0.0223 |
| 16777198 | 51084 | CRYL1 | crystallin, lambda 1 | -0.919 | 0.0489 |
| 16979432 | 79625 | NDNF | neuron-derived neurotrophic factor | -0.917 | 0.0334 |
| 17104521 | 54857 | GDPD2 | glycerophosphodiester phosphodiesterase domain containing 2 | -0.910 | 0.0104 |
| 16714618 | 288 | ANK3 | ankyrin 3, node of Ranvier (ankyrin G) | -0.909 | 0.0116 |
| 16675763 | 5317 | PKP1 | plakophilin 1 | -0.902 | 0.0320 |
| 16815925 | 92017 | SNX29 | sorting nexin 29 | -0.889 | 0.0259 |
| 16985751 | 11042 | SMA5 | glucuronidase, beta pseudogene | -0.881 | 0.0082 |
| 16766051 | 9924 | PAN2 | PAN2 poly(A) specific ribonuclease subunit | -0.881 | 0.0181 |
| 16752715 | 4035 | LRP1 | low density lipoprotein receptor-related protein 1 | -0.866 | 0.0264 |
| 16872982 | 27076 | LYPD3 | LY6/PLAUR domain containing 3 | -0.859 | 0.0493 |
| 16789484 | 122622 | ADSSL1 | adenylosuccinate synthase like 1 | -0.856 | 0.0338 |
| 16836624 | 406991 | MIR21 | microRNA 21 | -0.848 | 0.0430 |
| 16780664 | 84899 | TMTC4 | transmembrane and tetratricopeptide repeat containing 4 | -0.846 | 0.0208 |
| 16658023 | 9651 | PLCH2 | phospholipase C, eta 2 | -0.831 | 0.0116 |
| 16686060 | 6513 | SLC2A1 | solute carrier family 2 (facilitated glucose transporter), member 1 | -0.830 | 0.0223 |
| 16997046 | 100049076 | GUSBP9 | glucuronidase, beta pseudogene 9 | -0.821 | 0.0185 |
| 16781573 | 283 | ANG | angiogenin, ribonuclease, RNase A family, 5 | -0.813 | 0.0223 |
| 16709201 | 27250 | PDCD4 | programmed cell death 4 (neoplastic transformation inhibitor) | -0.811 | 0.0086 |
| 16776117 | 5095 | PCCA | propionyl CoA carboxylase, alpha polypeptide | -0.810 | 0.0202 |
| 16756310 | 255394 | TCP11L2 | t-complex 11, testis-specific-like 2 | -0.802 | 0.0223 |
| 16996971 | 100049076 | GUSBP9 | glucuronidase, beta pseudogene 9 | -0.798 | 0.0122 |
| 16974779 | 166647 | GPR125 | G protein-coupled receptor 125 | -0.794 | 0.0202 |
| 16932130 | 5625 | PRODH | proline dehydrogenase (oxidase) 1 | -0.792 | 0.0363 |
| 17104471 | 347517 | RAB41 | RAB41, member RAS oncogene family | -0.769 | 0.0161 |
| 16808326 | 9677 | PPIP5K1 | diphosphoinositol pentakisphosphate kinase 1 | -0.768 | 0.0187 |
| 16854316 | 114876 | OSBPL1A | oxysterol binding protein-like 1A | -0.768 | 0.0327 |
| 17061972 | 154743 | C7orf60 | chromosome 7 open reading frame 60 | -0.763 | 0.0263 |
| 16909958 | 8864 | PER2 | period circadian clock 2 | -0.756 | 0.0430 |
| 17072718 | 5820 | PVT1 | Pvt1 oncogene (non-protein coding) | -0.752 | 0.0430 |
| 16880176 | 400954 | EML6 | echinoderm microtubule associated protein like 6 | -0.749 | 0.0315 |
| 16833411 | 6362 | CCL18 | chemokine (C-C motif) ligand 18 (pulmonary and activation-regulated) | -0.745 | 0.0238 |
| 16723393 | 283267 | LINC00294 | long intergenic non-protein coding RNA 294 | -0.744 | 0.0035 |
| 17067170 | 665 | BNIP3L | BCL2/adenovirus E1B 19kDa interacting protein 3-like | -0.743 | 0.0258 |
| 16751190 | 25840 | METTL7A | methyltransferase like 7A | -0.737 | 0.0305 |
| 16756447 | 121551 | BTBD11 | BTB (POZ) domain containing 11 | -0.736 | 0.0327 |
| 16931361 | 55020 | TTC38 | tetratricopeptide repeat domain 38 | -0.730 | 0.0181 |
| 16942935 | 55279 | ZNF654 | zinc finger protein 654 | -0.721 | 0.0037 |
| 16769514 | 55198 | APPL2 | adaptor protein, phosphotyrosine interaction, PH domain and leucine zipper containing 2 | -0.720 | 0.0278 |
| 16990572 | 153769 | SH3RF2 | SH3 domain containing ring finger 2 | -0.714 | 0.0496 |
| 16748111 | 144571 | A2M-AS1 | A2M antisense RNA 1 (head to head) | -0.711 | 0.0383 |
| 16945365 | 339942 | H1FX-AS1 | H1FX antisense RNA 1 | -0.709 | 0.0459 |
| 17014869 | 80069 | LINC00574 | long intergenic non-protein coding RNA 574 | -0.704 | 0.0446 |
| 17070825 | 286144 | TRIQK | triple QxxK/R motif containing | -0.697 | 0.0399 |
| 16977711 | 5602 | MAPK10 | mitogen-activated protein kinase 10 | -0.688 | 0.0275 |
| 16933437 | 4330 | MN1 | meningioma (disrupted in balanced translocation) 1 | -0.686 | 0.0259 |
| 17105284 | 1730 | DIAPH2 | diaphanous-related formin 2 | -0.682 | 0.0259 |
| 16706023 | 317662 | FAM149B1 | family with sequence similarity 149, member B1 | -0.679 | 0.0447 |
| 16977579 | 84142 | FAM175A | family with sequence similarity 175, member A | -0.679 | 0.0446 |
| 16929148 | 91445 | RNF185 | ring finger protein 185 | -0.678 | 0.0115 |
| 16704094 | 399746 | ACTR3BP5 | ACTR3B pseudogene 5 | -0.674 | 0.0489 |
| 16764941 | 3852 | KRT5 | keratin 5 | -0.671 | 0.0097 |
| 17088589 | 11064 | CNTRL | centriolin | -0.668 | 0.0339 |
| 16827410 | 84080 | ENKD1 | enkurin domain containing 1 | -0.658 | 0.0250 |
| 17112170 | 100302692 | FTX | FTX transcript, XIST regulator (non-protein coding) | -0.657 | 0.0114 |
| 16668170 | 1952 | CELSR2 | cadherin, EGF LAG seven-pass G-type receptor 2 | -0.647 | 0.0438 |
| 16810585 | 80119 | PIF1 | PIF1 5'-to-3' DNA helicase | -0.646 | 0.0125 |
| 16844137 | 84961 | FBXL20 | F-box and leucine-rich repeat protein 20 | -0.646 | 0.0306 |
| 16813565 | 100507311 | LOC100507311 | uncharacterized LOC100507311 | -0.643 | 0.0389 |
| 16777185 | 2706 | GJB2 | gap junction protein, beta 2, 26kDa | -0.642 | 0.0428 |
| 16733643 | 112936 | VPS26B | vacuolar protein sorting 26 homolog B (S. pombe) | -0.638 | 0.0380 |
| 16698356 | 5287 | PIK3C2B | phosphatidylinositol-4-phosphate 3-kinase, catalytic subunit type 2 beta | -0.638 | 0.0275 |
| 16935455 | 5372 | PMM1 | phosphomannomutase 1 | -0.619 | 0.0202 |
| 17089593 | 51117 | COQ4 | coenzyme Q4 | -0.605 | 0.0298 |
| 16829922 | 50488 | MINK1 | misshapen-like kinase 1 | -0.605 | 0.0499 |
| 17082676 | 51160 | VPS28 | vacuolar protein sorting 28 homolog (S. cerevisiae) | -0.603 | 0.0223 |
| 16690343 | 10451 | VAV3 | vav 3 guanine nucleotide exchange factor | -0.602 | 0.0376 |
| 16704614 | 653145 | ANXA8 | annexin A8 | -0.600 | 0.0335 |
| 16938271 | 8850 | KAT2B | K(lysine) acetyltransferase 2B | -0.599 | 0.0253 |
| 17112376 | 254065 | BRWD3 | bromodomain and WD repeat domain containing 3 | -0.590 | 0.0459 |
| 16677425 | 1063 | CENPF | centromere protein F, 350/400kDa | -0.590 | 0.0440 |
| 16851749 | 1830 | DSG3 | desmoglein 3 | -0.585 | 0.0223 |
| 16872039 | 1891 | ECH1 | enoyl CoA hydratase 1, peroxisomal | -0.562 | 0.0377 |
| 16713762 | 728113 | ANXA8L1 | annexin A8-like 1 | -0.557 | 0.0447 |
| 16659545 | 114827 | FHAD1 | forkhead-associated (FHA) phosphopeptide binding domain 1 | -0.551 | 0.0472 |
| 16873487 | 339344 | MYPOP | Myb-related transcription factor, partner of profilin | -0.538 | 0.0131 |
| 16806968 | 440270 | GOLGA8B | golgin A8 family, member B | -0.528 | 0.0333 |
| 16744949 | 6330 | SCN4B | sodium channel, voltage-gated, type IV, beta subunit | -0.524 | 0.0305 |
| 16955064 | 7086 | TKT | transketolase | -0.502 | 0.0440 |
| 16665373 | 10207 | INADL | InaD-like (Drosophila) | -0.501 | 0.0487 |
| 17001901 | 2196 | FAT2 | FAT atypical cadherin 2 | -0.491 | 0.0305 |
| 16665566 | 5236 | PGM1 | phosphoglucomutase 1 | -0.466 | 0.0440 |
| 17004612 | 1832 | DSP | desmoplakin | -0.458 | 0.0430 |
| 16891132 | 79137 | FAM134A | family with sequence similarity 134, member A | -0.420 | 0.0459 |

RMA normalized microarray data was fitted to a linear model and initial statistics were determined using an empirical Bayesian model. Multiple testing comparisons were adjusted using Benjamini and Hochberg (aka FDR). Probes with an adjusted p-value <0.05 were considered statistically significant. The Affymetrix ProbeID, EntrezID, HUGO gene symbol, gene name, log2 fold change, and adjusted p-value are shown for the 116 genes lower in cisplatin ALDH^high^CD44^high^ vs. control ALDH^high^CD44^high^.
